# Pharmaceuticals and personal care products in Tunisian hospital wastewater: occurrence and environmental risk

**DOI:** 10.1007/s11356-023-31220-1

**Published:** 2023-12-08

**Authors:** Emna Nasri, Ana Cristina Soler de la Vega, Carlos Barata Martí, Hedi Ben Mansour, Maria Silvia Diaz-Cruz

**Affiliations:** 1 Research Unit of Analysis and Process Applied to the Environmental e APAE Higher Institute of Applied Sciences and Technology Mahdia, University of Monastir, Monastir, Tunisia; 2 Laboratory of Biotechnology and Bio-Monitoring of the Environment and Oasis Ecosystems, Department of Life Sciences, Faculty of Sciences of Gafsa, Sidi Ahmed Zarroug University Campus, 2112 Gafsa, Tunisia; 3 Department of Environmental Chemistry, Institute of Environmental Assessment and Water Research (IDAEA), Spanish Council for Scientific Research (CSIC), Jordi Girona 18-26, E-08034 Barcelona, Spain

**Keywords:** Contaminants of emerging concern, Hospital emissions, Liquid chromatography-mass spectrometry, Ecotoxicity, Hazard quotient

## Abstract

**Abstract:**

Despite concerns about the potential risk associated with the environmental occurrence of pharmaceuticals and personal care products (PPCPs), few studies address the emissions of hospitals to aquatic compartments. We examined within a 3-month sampling period the occurrence and environmental risk of PPCPs in seven Tunisian hospital wastewaters. From personal care products, UV filters, main metabolites, and benzotriazoles were quantified, with benzophenone 3 (oxybenzone, BP3) and benzotriazole (BZT) the most frequently found (71%) at median concentrations in the range 2.43 ± 0.87 ngL^−1^–64.05 ± 6.82 ngL^−1^ for BP3 and 51.67 ± 1.67 ngL^−1^–254 ± 9.9 ngL^−1^ for BZT. High concentrations were also found for 4-hydroxybenzophenone (4HB) (221 ± 6.22 ngL^−1^), one of the main metabolites of BP3. The antibiotics ofloxacin and trimethoprim, the anti-inflammatory acetaminophen, the antiepileptic carbamazepine, and the stimulant caffeine were present in all the wastewaters. The highest median concentration corresponded to acetaminophen, with 1240 ± 94 mgL^−1^ in Tunis Hospital, followed by ofloxacin with 78850 ± 39 μgL^−1^ in Sousse Hospital. For ecotoxicity assessment, acute toxicity was observed for *Daphnia magna* and *Vibrio fischeri*. The toxicity data were used in a hazard quotient (HQ) approach to evaluate the risk posed by the target PPCPs to aquatic organisms. The calculated HQs revealed that marbofloxacin (234 for *V. fischeri*), enrofloxacin (121 for *D. magna*), and BZT (82.2 for *D. magna* and 83.7 for *V. fischeri*) posed the highest risk, concluding that potential risk exists toward aquatic microorganisms. This study constitutes the first monitoring of UV filters in Tunisian hospital effluents and provides occurrence and toxicity data of PPCPs for reference in further surveys in the country.

**Graphical Abstract:**

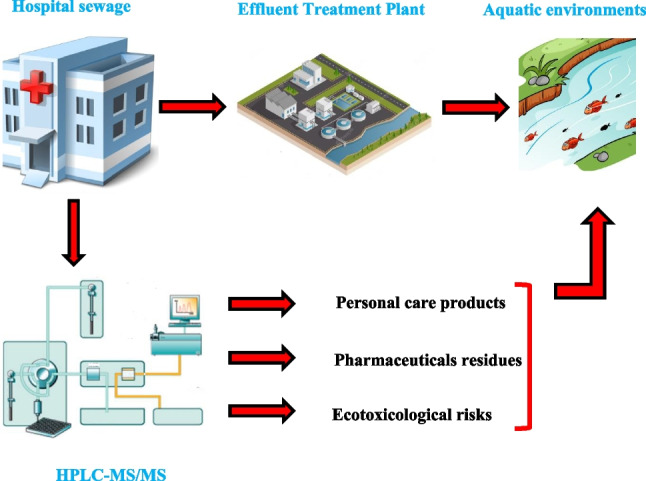

**Supplementary Information:**

The online version contains supplementary material available at 10.1007/s11356-023-31220-1.

## Introduction

In recent decades, pharmaceutical innovation has been key to improving quality and increasing life expectancy. If the pharmaceuticals marketed between the 80s and 2000 were responsible for more than 40% of the increase in life expectancy worldwide, in the first decade of this century, the percentage has increased to 73% (Lichtenberg 2022). Besides, their use has allowed the eradication of diseases that caused a high rate of mortality and/or disability or have made them considered chronic. The pharmaceutical industry in response to increased demand has been growing rapidly all over the world (Arvaniti et al. 2023). Inevitably, greater consumption implies a greater discharge of pharmaceuticals and their metabolites into wastewater. We consume many pharmaceuticals at home, at work, or in study centers. However, there are many and, in some cases, very specific medications that are consumed in health centers, mainly hospitals.

Pharmaceuticals can reach the aquatic environment via wastewater from the drug manufacturing industry, hospitals (HWW), and urban wastewater (Omuferen et al. 2022). Analgesics and antibiotics are those found the most (Verlicchi et al. 2010; Oliveira et al. 2017a, 2017b). Several studies have reported the occurrence of antibiotics in hospital effluents all over the world. However, not only pharmaceuticals but other chemicals included in daily-use products can be found in hospital wastewater, for instance, personal care and hygiene products (PCPs).

Despite the particular presence of biologically active residues of the compounds or their metabolism products, HWW is usually discharged into public sewage networks. It is ultimately treated in municipal wastewater treatment plants (WWTPs), together with wastewater from other origins. Over the last two decades, many studies have demonstrated the incomplete removal of thousands of pharmaceuticals and personal care products (PPCPs) at WWTPs (Santos et al. 2013; Pérez-Lemus et al. 2019**;** Afsa et al. 2020). Conventional wastewater treatments were designed, at best, to remove N, P, and microbiological contamination (pathogens). However, hospital discharge contains large amounts of hazardous compounds and microbial agents. The existence of chemicals in HWW (disinfectants, X-ray contrast agents, and antibiotics, among many other PPCPs) has become one of the increasing concerns of the scientific community (Mazzitelli et al. 2018). Many of these compounds are recalcitrant to conventional wastewater treatments and, thus, end up in surface waters (Sadutto et al. 2020). Unfortunately, they impact aquatic organisms and humans (via the food chain) even at very low concentrations (Al Aukidy et al. 2014; Carraro et al. 2016). Most HWW streams are directly discharged into the sewage system without prior treatment. Hospitals generate between 200 and 1200 L of wastewater per bed per day, whereas domestic wastewater generation is substantially lower, in the range of 100–400 L per person per day (Al Aukidy et al. 2014; Carraro et al. 2016; Kumari et al. 2020). Moreover, according to Verlicchi et al. (2010), it has been demonstrated that the physicochemical parameters of HWWs can be 2–3-fold greater than those of an urban effluent. Similarly, the potential toxicity can be 5–15-fold higher in HWW than in urban wastewater (Kumari et al. 2020). HWW can pose three types of risk, namely, infectious, radioactive, and toxic. Antibiotics are among the therapeutic groups that are more frequently detected in HWW (Verlicchi et al. 2012; Santos et al. 2013). Hospitals are hotspots for specialized pharmaceuticals entering the aquatic environment. Up to 90% of orally administered pharmaceuticals are excreted as active substances in the feces and urine of patients (BIO Intelligence Service 2013).

The concentration and impacts of PPCPs in the environment depend on a combination of factors, including their dosage, toxicity, degradation, persistence, and mobility (Afsa et al. 2021; Queen et al. 2022a, 2022b). An additional stumbling block to fully understanding the hazard posed by these chemicals to the environment is that the outcomes of a survey performed in a country cannot be extrapolated to others. This fact is attributed to the different regulations and patients in each country. It is, therefore, necessary to conduct monitoring campaigns in every particular country.

In the present study, we investigated the occurrence of the highly consumed PPCPs and their transformation products in effluents from seven hospitals included in the network of public hospitals in Tunisia. The chemical characterization was carried out by high-performance liquid chromatography coupled to tandem mass spectrometry (HPLC-MS/MS). Furthermore, to assess the correlation between the occurrence of PPCPs in the hospital effluents and their potential ecotoxicological effects, the toxicity of the HWW effluents was evaluated against two bioindicator species extensively used to estimate the ecotoxicity of chemicals, i.e., the planktonic crustacean *Daphnia magna* and the bioluminescent bacteria *Vibrio  fischeri*. The data generated allowed us to perform a preliminary environmental risk assessment through the estimation of hazard quotients (HQs).

## Materials and methods

### Chemicals and reagents

Benzophenone 3 (BP3), benzophenone 1 (BP1), benzophenone 2 (BP2), 4-hydroxy benzophenone (4HB), 4,4′-dihydroxy benzophenone (4DHB), 2,2′-dihydroxy-4-methoxy benzophenone (DHMB), 3-(4-methyl benzylidene)camphor (4-MBC), ethyl-4-p-aminobenzoic acid (EtPABA), ethyl hexyl dimethyl-4-p-aminobenzoic acid (ODPABA), 1H-benzotriazole (BZT), 5-methyl-(1H-benzotriazole) (MeBZT), 5,6-dimethyl-1H-benzotriazole (DMBZT), 2-(2H-benzotriazol-2-yl)-p-cresol (UVP), clarithromycin (CLR), enoxacin (ENO), enrofloxacin (ENR), flumequine (FLU), marbofloxacin (MAR), nalidixic acid (NAX), oxolinic acid (OXO), oxytetracycline (OTC), succynil-sulfathiazole (SST), sulfadiazine (SDZ), N4-acetylsulfadiazidine (acSDZ), sulfamerazine (SMR), N4-acetylsulfamerazine (acSMR), N4-acetylsulfamethazine (acSMZ), sulfamethoxazole (SMX), N4-acetylsulfamethoxazole (acSMX), sulfamethoxypyridazine (SMP), sulfapyridine (SPY), N4-acetylsulfapyridine (acSPY), sulfaquinoxaline (SQX), sulfathiazole (STZ), sulfisomidin (SSM), sulfadimethoxine (SDM), sulfanitran (SNT), sulfabenzamide (SBD), trimethoprim (TMP), penicillin V (PEN), pipemidic acid (PIP), naproxen (NAP), carbamazepine (CBZ), carbamazepine 10,11-epoxy (CBZ-epoxy), ofloxacin (OFL), and caffeine (CAF) of the highest purity were obtained from Sigma–Aldrich (Steinheim, Germany), Fluka (Seelze, Germany), and Toronto Research Chemicals (Toronto, ON, Canada).

Acetonitrile (ACN), methanol (MeOH), formic acid, and HPLC-grade water were provided by Merck (Darmstadt, Germany). Ar and N_2_ were purchased from Air Liquide (Barcelona, Spain). Nylon membrane and glass fiber filters were obtained from Whatman International Ltd. (Maidstone, England). The individual stock standard solutions were prepared in MeOH and stored in the dark at −20 °C. Toxicological kits for *D. magna* (Daphtoxkit F) were purchased from Microbiotests (Gent, Belgium). Bacteria *V. fischeri* (NRRL B-1117) was supplied as a freeze-dried reagent (BioFix^®^ Lumi, Macherey-Nagel, Germany) and stored at −20 °C until re-hydration before testing. A stereomicroscope SZT from VWR (Llinars del Vallés, Spain) was used for the *D. magna* tests, and for the *V. fischeri* tests a Model 500 analyzer from SDI (USA).

### Sampling

**Fig. 1:**
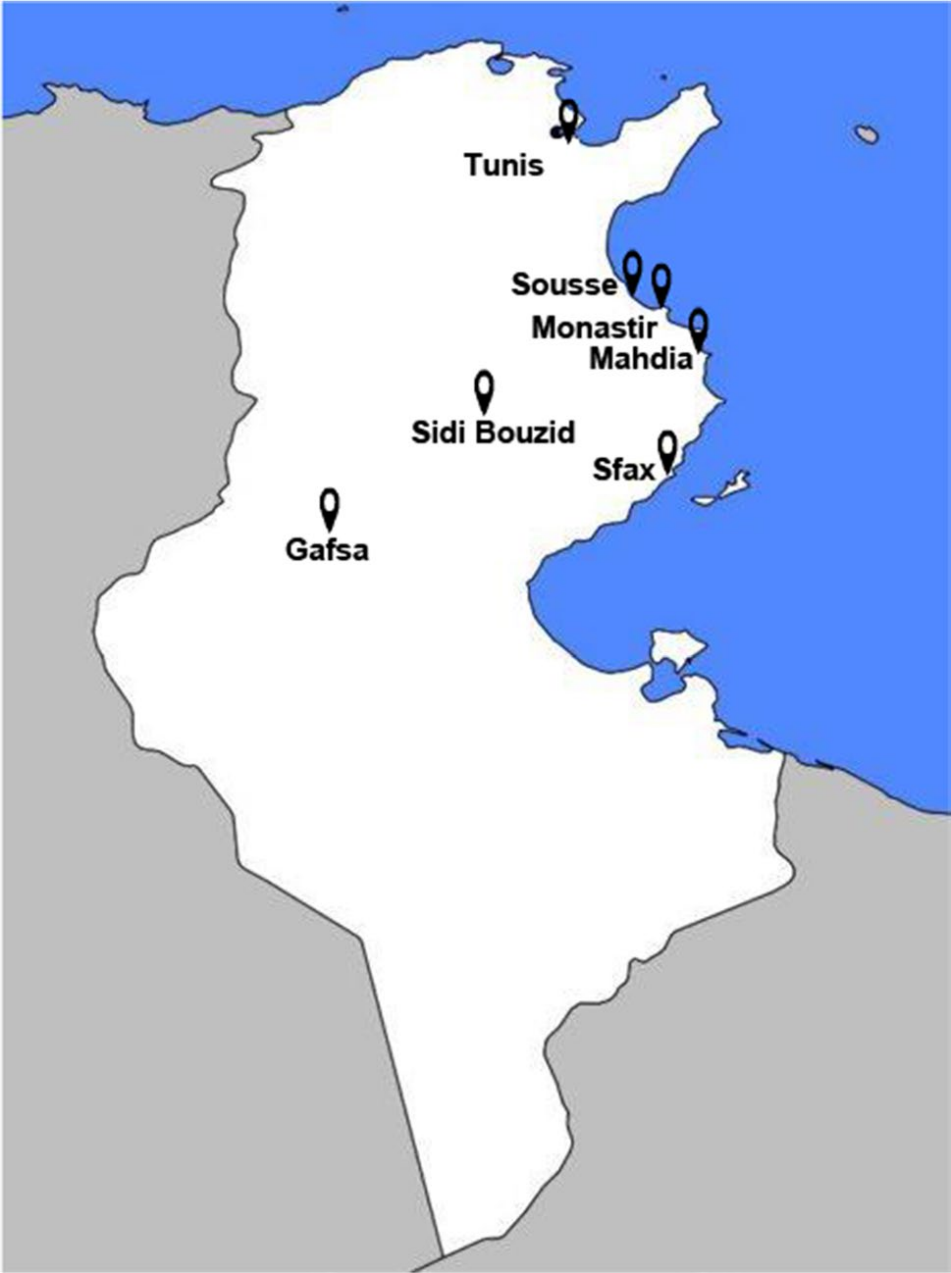
Area of study and sampling locations of the seven selected hospitals in Tunisia

Table 1 lists the names and characteristics of the seven selected hospitals in the Tunisian municipal network. The sampling points were located on the main drain, receiving the discharges of the wastewater from the selected hospitals (Fig. 1). These wastewaters are not treated at source and are composed of the contributions of wastes from the following departments: general medicine, surgery, intensive care, maternity, gynecology, oncology, psychiatry, rheumatology, hematology, hepatic-gastroenterology, radiology, and laboratories. According to the “State of the Environment in Tunisia” report published in 2022 by the Tunisian Forum for Economic and Social Rights, and National Sanitation Office (ONAS 2023) in Tunisia, around 70% wastewater is discharged directly into the environment without undergoing adequate treatment. Hospital effluents are directly released into public sewerage systems without pre-treatment and then introduced into municipal WWTPs, where they are co-treated with other effluent (Afsa et al. 2021; Beltifa et al. 2019). At the time of sampling in each of the selected hospitals, we confirmed that none of them had any wastewater treatment system in place.

**Table 1:** Location and characteristics of the seven Tunisian hospitals selected for the study. Data were obtained from the report issued by the Tunisian Ministry of Health in 2013. In all the cases, the hospital wastewater effluent was collected

Hospital (location)	GPS coordinates	Consultations	Emergency cases	No. of beds
Charles Nicolle Univ. Hospital (Tunis)	Latitude 36.802247|longitude 10.161222	231538	112470	1049
Farhat Hached Univ. Hospital (Sousse)	Latitude 35.82961|longitude 10.627749	210854	128691	670
Fattouma Bourguiba Univ. Hospital (Monastir)	Latitude 35.76774|longitude 10.83637	196264	116807	918
Hedi Chaker Univ. Hospital (Sfax)	Latitude 34.740725|longitude 10.750304	172101	35174	821
Tahar Sfar Univ. Hospital (Mahdia)	Latitude 35.510075|longitude 11.032643	124613	65859	389
Regional Hospital (Gafsa)	Latitude 34.420007|longitude 8.796143	67256	127611	327
Regional Hospital (Sidi Bouzid)	Latitude 35.023948|longitude 9.465184	28717	5198	441

Sampling was conducted from February to April 2019 at seven selected hospitals across Tunisia. Wastewater was sampled in triplicate by maintenance staff from the facilities at 3-hour intervals daily, spanning from 8 am to 8 pm, for a month, utilizing 1 L sterile borosilicate amber glass bottles. The samples were promptly refrigerated and transported to the laboratory for freezing at −20 °C in the absence of light. Upon thawing, all samples were proportionally combined to create a bulk sample per month per hospital. An aliquot of 1 L from these integrated samples was extracted for subsequent analysis. In total, twenty-one hospital wastewater samples were analyzed.

### Sample pre-treatment

The methods by Gago-Ferrero et al. (2013), García-Gil et al. (2018), and Molins-Delgado et al. (2017) were used as a basis for the determination of the target PPCPs in the wastewater samples. Briefly, samples were filtered through a 0.45 μm pore nylon membrane filter and a 0.2 μm pore glass fiber filter to remove all suspended particulate matter to analyze the dissolved fraction. After filtration, 50 μL of the internal standards mix solution at 50 ng/mL was added to 50 mL of the water samples for further online solid-phase extraction (SPE). The automated online SPE-HPLC Symbiosis Pico (Emmen, Holland) equipment was used to purify and pre-concentrate the samples. For their online SPE extraction, 5 mL of the filtered HWW samples, aqueous standard mixture solutions, and blanks were loaded onto online SPE Oasis HLB cartridges from Waters (Milford, MA, USA). The trapped analytes were eluted to the HPLC analytical column through the mobile phase, which consisted of a mixture of HPLC-grade water and ACN (both 0.1% in formic acid).

### HPLC-MS/MS analysis

The mass spectrometry detector, a 4000 QTRAP™ MS/MS hybrid quadrupole-linear ion-trap (QqLIT), equipped with a Turbo Ion Spray source from Applied Biosystems-Sciex (Foster City, Ca, USA) was connected in series with the Symbiosis Pico and controlled by the Analyst software v. 1.4.2. (Applied Biosystems-Sciex). The chromatographic separation was performed on a Merck’s Hibar Purosher® STAR® HR R-18 ec (125 mm × 2.0 mm, 5 μm) analytical column. The total run time was 25 min. MS/MS detection was operated in both positive and negative electrospray ionization (ESI) modes. Data acquisition was carried out in selected reaction monitoring (SRM) mode. General operational conditions were as follows: capillary voltage, 5000 V; source temperature, 700 °C; curtain gas, 30 psi; ion source gas 1.50 psi; ion source gas 2.60 psi; and entrance potential, 10 V. Specific conditions for each analyte can be found in Table 1S of the Supplementary Information. Quantification was carried out by isotope dilution. For the positive presence of a compound in a measured sample, we followed the European Commission guidelines (Council of the European Communities 2002). Two transitions were selected; the most abundant was used for quantification (SRM1) and the second was used for identity confirmation (SRM2). The chromatographic retention time of the target PPCPs in the sample must match that of the PPCPs standards with a margin of ±2%, and their SRM1/SRM2 ratio cannot deviate by >20–50% from the corresponding ratio in the standard solutions. Instrument control and data acquisition and evaluation were performed with the Analyst 1.4.2 software from Applied Biosystems/MDS Sciex and the Symbiosis from the Symbiosis Pico for Analyst software.

Recovery rates were quite variable, as usual for multi-residue analytical methods including compounds with very different physicochemical properties, from 45.5 to 122%. The method provided a wide linearity range being the calibration range between 1 and 700 ngmL^−1^. For all the compounds precision was quite good, with RSD usually below 15%. The method limits of detection (LODs) were in the range of 0.10–1.21 ngL^−1^ and the method limits of quantification (LOQs) varied from 0.32 to 4.0 ngL^−1^. Specific values for the target compounds are listed in Table 2S.

### QA/QC in chemical analysis

The quality assurance (QA) and quality control (QC) criteria applied comprised several measures.

To ensure the reliability of the results, a series of preventive actions were taken to avoid cross-contamination of the samples and standards, including the use of gloves during the whole analytical process, the use of solvents and reagents exclusively employed for these analyses, the extensive cleaning treatment of all laboratory material washing with HPLC-grade water and solvents of different polarity (MeOH, dichloromethane, and acetone), and further heating at 350 °C overnight.

Analyses of laboratory blanks (pure solvents and reagents to test for instrument contamination and to prevent memory effects and flux of potential retained compounds) were performed to find potential background levels. The daily set of samples for analysis was processed along with a blank extract to avoid contamination during the extraction process leading to false positives. Ten matrix-matched calibration standards were freshly prepared to test both linearity and sensitivity along the working range of concentrations. To check for potential instrumental drift in response factors, a standard mixture solution (as control) was included in all the analysis sequences. Quantification was based on isotope dilution, using the corresponding ^13^C and deuterium labeled isotopic standard and measured as the area of the peaks obtained. The same procedure was applied to both the samples and the ten matrix-matched calibration standard solutions used. This approach overcomes the matrix effects expected in the complex matrix as does HWW.

## Acute toxicity estimation

### *Daphnia magna* assays

Individuals of a single clone of *D. magna* were maintained in 120 mL of ASTM hard water in 150 mL screw-top glass jars, with the addition of a standard organic extract. The daphnids were fed daily with *Chlorella vulgaris* Beijerinck 201 (5 × 105 cells mL^−1^, corresponding to 1.8 mgL^−1^), the photoperiod was set to 14-h light and 10-h dark cycle, and the temperature was kept to 20 ± 1 °C. Acute toxicity tests were conducted, according to the conditions previously described by Molins-Delgado et al. (2016). Neonates (<24 holds) in groups of 5 were transferred using a disposable Pasteur pipette from the vessels to a Petri dish (10 mL). ASTM water was used as a diluent. The individuals were exposed to HWW at 6 proportions: 3%, 6%, 12%, 25%, 50%, and 100 %. Petri dishes were illuminated with a constant light (3000 lux) and kept at 22 °C. The number of immobilized neonates was monitored at 24 and 48 h and related to the dilution of HWW, thus allowing us to determine the 24 h and 48 h LC50 values. Two bioassays per HWW proportion were carried out, with two replicates each.

### *Vibrio fischeri* assays

The bioassay on bacterial luminescence was carried out with an analyzer controlled by the software Microtox Omni (SDI), equipped with a 30-well incubator chamber set to 15 °C. *V. fischeri*’s acute toxicity test was conducted based on the ISO 11348-3 guideline. Two replicate tests were carried out per sample. A culture medium was prepared consisting of water with 3% NaCl. Standard solutions and HWW were handled according to the Microtox manual. Vials containing the bacteria were stored at 20 °C. We prepared as many cells as blanks, dilutions, and replicates added 1 mL of the medium to each cell, and inserted the cells in each well. The bacteria were reconstituted in 2 mL of the included reagent and placed in cells. Then, an aliquot of 10 μL of the bacteria was placed in a cell for measuring the absorbance. Once the absorbance was measured, 1 mL of the dilution of HWW was added to the corresponding cell. After 15 and 30 min, photometric measures of each cell were recorded, allowing the estimate of the EC_50_ values, i.e., the reduction in the light output of *V. fischeri* by 50%. All experiments were conducted in triplicate and repeated two times.

### Acute toxicity data evaluation

#### *Daphnia magna*

To obtain the response profiles and LC50 of each HWW, the percent of inhibition (I) was calculated following Eq. 1 and related to the different dilution of each HWW using the Hill regression model, as expressed in Eq. 2, and the curve fitting tools of the Graph Pad Prism software.

1 $$I=\frac{D_i}{D_0}\times 100$$

where *D*_0_ is the number of initial neonates and *D*_*i*_ is the number of immobilized ones:

2 $$I=B+\frac{\left(T-B\right)}{\left(1+{10}^{\left(\left(\textrm{Log}{\textrm{EC}}_{50}-X\right)\ast H\right)}\right)}$$

where *T* is the top value of the curve, *B* is the lower parameter of the curve, LogEC_50_ is the logarithm of the median effect concentration, *X* is the logarithm of concentration (proportion of each HWW sample), and *H* is the Hill coefficient of the curve.

#### *Vibrio fischeri*

Bioluminescence was determined by absorbance measurements. To estimate the incidence rates, Eq. 3 was applied:

3 $$I=\frac{{\textrm{ABS}}_0-{\textrm{ABS}}_t}{{\textrm{ABS}}_t}$$

where *I* is the percent of inhibition, ABS_*t*_ is the absorbance at the time of the analysis, and ABS_0_ is the initial absorbance. Inhibition rates were then correlated with the different proportions of the HWW described by Eq. 2. Toxicity was estimated by calculating the toxicity units (TU) (Tamura et al. 2017) according to Eq. 4

4 $$\textrm{TU}=\frac{100}{{\textrm{ECx}}_{\textrm{HWW}}}$$

where ECx_HWW_ is the X% effect concentration of the HWW sample. A value of TU < 1 indicates a nontoxic effect, whereas TU > 1 corresponds to a toxic effect.

### Toxicity estimation

For a hazard assessment, there are several approaches to calculating a hazard (or risk) quotient (U.S. Environmental Protection Agency ((USEPA 2018); Dussault et al. 2008; European Medicines Agency (EMEA 2006); European Commission 2003).

The definition of a hazard or risk quotient is the ratio of the potential exposure to a substance and the level at which no adverse effects are expected (USEPA 2018). Hazard quotient equations require at least two parameters, i.e., a measured environmental concentration (MEC) and a toxicity endpoint, for instance, the lethal concentration for 50% of the population (LC50) or the concentration of a chemical at which adverse effects are observed in 50% of the population (EC50, sub-lethal endpoint). A hazard quotient (HQ) calculation following the protocol specified by the USEPA guidance for pesticides and other chemicals (USEPA 2005) compares the MEC to an acute toxicity endpoint (e.g., LC50) Thus, an HQ is a screening tool that generates measures of levels of concern, though this method does not provide probability-based information on risk. The equation for calculation is HQ = (MEC) / (organism’s EC50 or LC50 with 96 h or less of exposure to the toxicant).

The European Commission methodology has been adopted in the development of several ecological risk assessment guidelines (ECHA 2008; EMEA 2006; European Commission  2003). With this method, the actual measured environmental concentration, i.e., MEC, is compared to a derived known or Predicted No-Effect Concentration (PNEC) which is obtained by dividing the LC50, EC50, or NOEC by an uncertainty factor (UF). Thus, the HQ is expressed as the ratio (MEC)/(PNEC)(UF). For this HQ determination, for instance, a UF of 1000 was selected by Dussault et al. (2008) for the extrapolation of the EC50, LC50, or No Observable Effect Concentration (NOEC) values to estimate PNEC. However, the authors also estimated a second HQ by the incorporation of occurrence data from several studies, leading them to reduce the UF to 100, since these HQ estimates were then based on acute toxicity data from three or more taxa. When the NOEC is not known, but the Lowest Observable Effect Concentration (LOEC) is available, the LOEC is divided by 2 to estimate a NOEC (ECHA 2008).

In the present study, we adopted the concentration addition (CA) model to estimate the total toxicity of the WWTP samples as the contribution of each PPCPs. To this end, individual HQs were calculated following the EMEA guidelines (EMEA 2006). The HQ was calculated as the ratio between the median measured environmental concentration of component *i* (MEC_i_) (whereas maximum values were measured in our sampling from our analyses) and the predicted non-effect concentrations (PNEC_*i*_) of the *i* component extracted from the literature (Table 3S). Consequently, the HQ of each HWW sample was calculated by Eq. 5:

5 $$\textrm{HQ}\left(\textrm{HWW}\right)=\sum\nolimits_{i=1}^n\frac{\textrm{MECi}\ }{\textrm{PNECi}\ }$$

The PNEC estimated for acute exposures was derived from the EC50 values of each HWW using an uncertainty factor of 100 according to the OCDE 1995 guideline, as shown in Eq. 5.

6 $$\textrm{PNEC}=\frac{\textrm{EC}50}{100\ }$$

HQs > 1 indicate an ecotoxicological risk for the aquatic environment, 0.5 < HQs < 1 corresponds to scenarios with medium risk, and HQs < 0.5 indicate no ecotoxicological risk.

## QA/QC and statistical analysis in bioassays

As in the case of the chemical analyses, all material used was cleaned or disposable. The reagents and solvents used were of toxicity bioanalysis quality.

All statistical analyses were carried out by the R software version 4.2.2 (R Core Team version 4.2.2). The median values for the target PPCPs in the HWW were analyzed using one-way ANOVA (Analysis of variance). Tukey’s test was used to compare the median concentrations in wastewater at the different hospitals at *p* = 0.05. The correlation between the toxicity and other parameters was made using the Pearson correlation coefficient at *p* = 0.01.

## Results

**Fig. 2:**
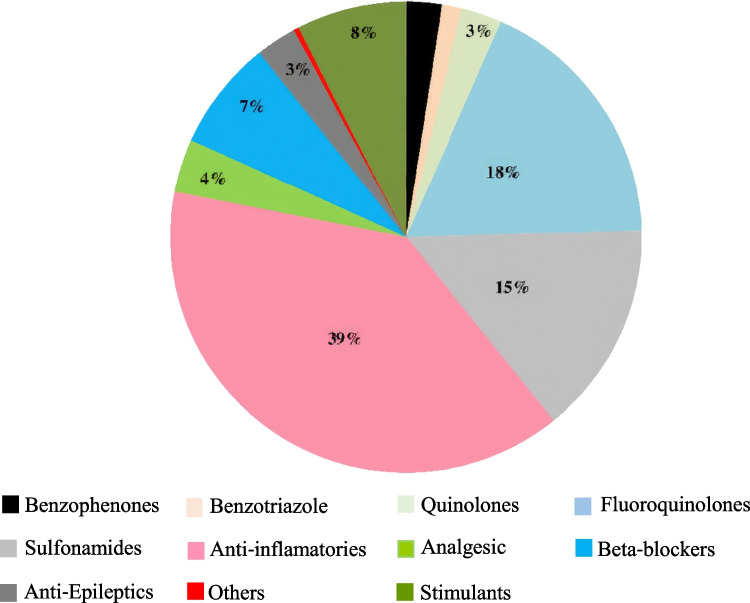
Distribution of pharmaceuticals and personal care products in the hospital wastewater (HWW) according to family groups

**Fig. 3:**
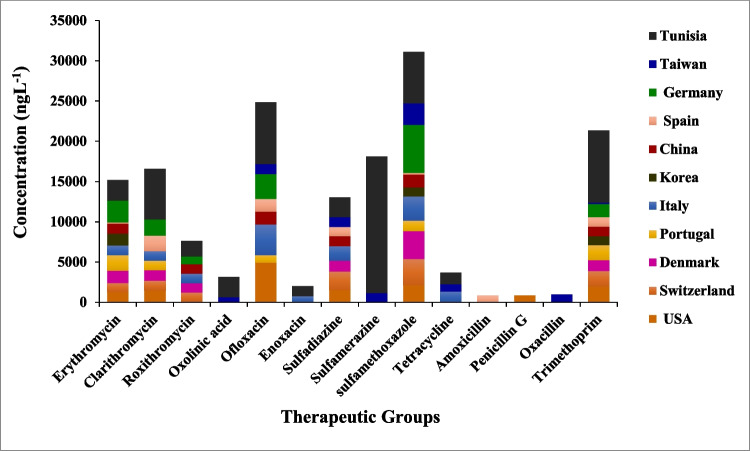
Occurrence of PPCPs in hospital effluents all over the world and in the present study (Tunisia)

The general quality parameters of the HWW from the seven selected hospitals in Tunisia can be found in Nasri et al. (2017). The main data are summarized in Table 4S of the Supplementary Information. Figure 2 shows the distribution of PPCPs measured in the analyzed HWW according to therapeutic classes, and Fig. 3 shows the mean concentrations reported in studies from other countries, as well as the results of the present study.

### Occurrence of personal care products

Table 2 lists the median concentrations corresponding to the compounds detected at least once. Concentrations of target PPCPs in each sample are compiled in Table 5S. Eight UV filters out of the 13 analyzed were detected. The benzophenones BP3 and BP2 along with their human metabolites were present at least in 1 sample out of the 21 HWW analyzed. BP3 was found in 71% of the samples. The concentrations of its human metabolites, BP1, 4HB, and 4DHB, suggested that BP3 is mainly metabolized and further excreted. In contrast, BP2 appeared to be less prone to human metabolism, being transformed to a lesser extent and found as the parent compound at quite high concentrations in 6 out of the 7 hospital effluents analyzed. The median maximum concentration detected for BP2 was 154.33 ± 4.16 ngL^−1^ in Monastir. One of the main metabolites of benzophenone-type UV filters is 4HB. Particularly high were the median concentrations of 4HB with a maximum of 221 ± 6.22 ngL^−1^ in Gafsa.

**Table 2:** Median values of concentrations, method limits of detection (LODs) and quantification (LOQs), and frequencies of detection (Freq) for the target Pharmaceuticals and Personal Care Products (PPCPs) in the hospital wastewater samples analyzed (ngL^−1^). n.d., not detected. Values with different letters as superindex indicate significant differences according to the Tukey test (*p* < 0.05).

	Median concentrations of PPCPs (ngL^−1^) in hospital Wastewaters							LOD (ngL^−1^)	LOQ (ngL^−1^)	Freq (%)
Compounds (acronym)	Gafsa	Monastir	Sousse	S. Bouzid	Sfax	Tunis	Mahdia			
Benzophenones										
Benzophenone 1 (BP1)	n.d.	n.d.	n.d.	11.66 ± 3.54^a^	n.d.	n.d.	n.d.	0.44	1.46	5
Benzophenone 2 (BP2)	n.d.	154.33 ± 4.16^a^	34 ± 5.14^c^	22 ± 1.45^a^	31 ± 7.84^b^	28 ± 1.53^b^	52.5 ± 4.13^b^	0.6	2.03	33
Benzophenone 3 (BP3)	24.17 ± 8.1^ab^	n.d.	24.9 ± 2.68^ab^	52.35 ± 9.69^ab^	2.43 ± 0.87^b^	3.16 ± 3.75^b^	64.05 ± 6.82^a^	0.18	0.6	71
4-Hydroxybenzophenone (4HB)	221 ± 6.22^a^	n.d.	n.d.	195 ± 5.6^b^	73 ± 8.94^b^	n.d.	n.d.	0.6	2	19
4-Dihydroxybenzophenone (4DHB)	n.d.	12 ± 2.34^a^	n.d.	n.d.	n.d.	13 ± 0.94^a^	107 ± 18.27^b^	1.18	3.95	14
Benzotriazoles										
1H-Benzotriazole (BZT)	51.67 ± 1.67^b^	254 ± 9.9^a^	169.8 ± 4.95^a^	78.5 ± 3.15^ab^	215 ± 8.79^ab^	177 ± 8.44^ab^	181.5 ± 8.56^ab^	0.33	1.12	71
Dimethylbenzotriazole (DMBZT)	n.d.	n.d.	204.33 ± 14.57^b^	n.d.	48 ± 3.46^a^	n.d.	n.d.	0.45	1.52	21
Methylbenzotriazole (MeBZT)	n.d.	n.d.	n.d.	n.d.	187 ± 5.28^a^	n.d.	n.d.	0.17	0.59	7
Macrolides										
Clarithromycin (CLR)	n.d.	7 ± 1.3^a^	264.5 ± 8.43^b^	17.3 ± 4.16^a^	4.78 ± 0.91^a^	7.18 ± 0.64^a^	n.d.	0.2	0.6	51
Quinolones										
Enoxacin (ENO)	83.67 ± 7.38^a^	81.33 ± 14.8^a^	n.d	67 ± 6.7^a^	26.33 ± 5.61^a^	n.d.	n.d.	0.1	0.3	19
Nalidixic acid (NAX)	n.d.	n.d.	1098 ± 8.94^a^	n.d.	4672 ± 46.17^b^	893 ± 17.8^a^	n.d.	0.18	0.62	36
Oxolinic acid (OXO)	18 ± 2.8^a^	24 ± 3.45^a^	n.d.	23 ± 4.8^a^	58.9 ± 4.7^b^	n.d.	8 ± 0.8^c^	0.13	0.45	71
Enrofloxacin (ENR)	n.d.	484.67 ± 6.45^a^	214 ± 8.29^a^	43 ± 0.7^b^	153.3 ± 5.85^a^	124 ± 8.29^a^	260 ± 6.3^a^	0.3	0.9	52
Pipemidic acid (PIP)	n.d.	n.d.	n.d.	31.67 ± 4.85^b^	99.67 ± 2.63^a^	143.3 ± 0.5^a^	252 ± 4.83^a^	0.6	2	33
Fluoroquinolones										
Marbofloxacin (MAR)	31.33 ± 4.27^b^	25 ± 5.7^b^	124 ± 2.65^ab^	559 ± 6.98^a^	3 ± 0.4^b^	n.d.	93 ± 4.17^ab^	0.7	2.4	43
Flumequine (FLU)	114 ± 7.8^b^	8 ± 3.1^a^	n.d.	16 ± 4.8^a^	n.d	n.d	n.d	0.21	0.70	14
Ofloxacin (OFL)	18800 ± 17.6^e^	43050 ± 64.84^bcd^	78850 ± 39.3^a^	23750 ± 51.3^de^	56900 ± 60^ab^	50050 ± 50.1^bc^	31850 ± 4.12^cde^	0.86	2.89	100
Tetracyclines										
Oxytetracycline (OTC)	60 ± 3.39^a^	3.33 ± 0.57^ab^	47.33 ± 2.44ab	n.d.	8.67 ± 0.5^ab^	11.67 ± 0.21^ab^	47.33 ± 2.44^ab^	0.26	0.88	38
Sulfonamides										
Sulfabenzamide (SBD)	n.d.	n.d.	n.d.	n.d.	4 ± 0.73^a^	n.d.	n.d.	0.2	0.6	5
Sulfadimethoxine (SDM)	n.d.	98 ± 3.71^b^	124 ± 27.1^a^	n.d.	n.d.	n.d.	n.d.	0.49	1.65	10
Sulfamerazine (SMR)	n.d.	160 ± 11.29^a^	n.d.	n.d.	n.d.	n.d.	n.d.	1.2	4	5
Sulfamethoxazole (SMX)	n.d.	58.33 ± 1.04^a^	153.67 ± 6.67^a^	n.d.	494.5 ± 15.6^b^	349.5 ± 17.28^b^	140 ± 4.18^a^	0.6	2	71
Sulfapyridine (SPY)	1342.63 ± 9.5^b^	254.5 ± 12.85^b^	320.67 ± 1.56^b^	215.17 ± 9.9^b^	1510 ± 6.15^b^	10990 ± 10^b^	476.17 ± 5.9^a^	0.35	1.18	93
Acetyl sulfapyridine (AcSPY)	n.d.	n.d.	n.d.	n.d.	195.5 ± 4.2^b^	1142 ± 6.37^a^	n.d.	0.25	0.85	29
Sulfadiazine (SDZ)	n.d.	n.d.	n.d.	n.d.	88.45 ± 1.55^b^	671.5 ± 9.6^a^	53.1 ± 4.6^b^	0.3	1.2	36
Anti-inflammatories										
Ketoprofen (KEP)	n.d.	n.d.	n.d.	n.d.	673.3 ± 25.17^a^	n.d.	1190 ± 24.02^b^	0.27	0.90	28
Mefenamic acid (MFA)	267.5 ± 5.5^b^	562 ± 9.14^b^	511.67 ± 9.67^b^	468 ± 12^b^	1129.33 ± 37.9^b^	4465 ± 21^a^	562 ± 13.3^b^	0.31	1.03	93
Acetaminophen (AMP)	838000 ± 5 0^ab^	759333 ± 50^ab^	495000 ± 66.6^b^	988500 ± 60^ab^	628000 ± 69^ab^	1240000 ± 94^a^	545100 ± 50.6^b^	0.19	0.63	100
Beta-blockers										
Atenolol (ATL)	7340 ± 25^bc^	3440 ± 29^c^	2155 ± 68.11^c^	19883.3 ± 75.4^a^	11666.7 ± 50.6^b^	11783.3 ± 17^b^	6546.67 ± 69.8^bc^	0.39	1.32	93
Anti-epileptics										
Carbamazepine (CBZ)	63.65 ± 9.4^d^	195.5 ± 8.76^b^	562 ± 9.94^a^	131.5 ± 9.5^cd^	324.5 ± 10.42^bc^	417.5 ± 10.6^ab^	381.5 ± 15.21^b^	0.28	0.94	100
Carbamazepine-10,11-epoxide (CBZ-epoxy)	n.d.	4.7 ± 1.4^a^	28.87 ± 0.96^a^	n.d.	13.98 ± 3^a^	n.d.	36.5 ± 1.1^b^	0.19	0.66	29
Others										
Trimethoprim (TMP)	53.7 ± 5.9^b^	72.17 ± 9.13^b^	58.5 ± 3.42^b^	39.25 ± 1.57^b^	175 ± 2.96^a^	29.15 ± 2.5^b^	81.53 ± 5.45^b^	0.36	1.21	100
Caffeine (CAF)	14350 ± 50^c^	26316.7 ± 60^b^	5331.67 ± 51.9^d^	22500 ± 10^b^	20550 ± 45.64^bc^	27466.7 ± 52^b^	38900 ± 23^a^	1.14	3.80	100

Benzotriazoles were present frequently (71%). Interestingly, the highest concentrations observed for the benzotriazole family corresponded to BZT (254 ± 9.9 ngL^−1^) in Monastir HWW. High values were also found in Sfax (215 ± 8.79 ngL^−1^) and Mahdia (181.5 ± 8.56 ngL^−1^).

### Occurrence of pharmaceuticals

Twenty-five out of the 37 pharmaceuticals investigated were detected in the HWW at a wide range of concentrations, from low ngL^−1^ to μgL^−1^, as listed in Table 2. Despite the wide application of antibiotics in numerous medical departments, compounds belonging to the cephalosporin and penicillin families, among the most prescribed antibiotics, were never detected.

In contrast, the stimulant caffeine showed the dominant concentration of the detected pharmaceuticals in all the HWW (up to 38900 ± 23 ngL^−1^ median concentration in Mahdia). Acetaminophen (paracetamol) is one of the pharmaceuticals that may be formulated together with caffeine in some medicines (Weigel et al. 2002); in this study, acetaminophen was found in all the samples and particularly at high concentrations in Tunis (maximum 1240000 ± 94 ngL^−1^). Likewise, caffeine is ubiquitous and highly concentrated, since besides being included in many pharmaceuticals, it is also present in many beverages, such as coffee, that are usually highly consumed at hospitals by patients and relatives and by medical and maintenance personnel.

Ofloxacin, nalidixic acid, and enrofloxacin were frequently detected (100%, 36%, and 52%, respectively) at concentrations up to 78850 ± 39.3 ngL^−1^ (Sousse).

Conversely, lower concentrations of macrolide antibiotics were detected. Only clarithromycin could be measured, in the range from 7 ± 1.3 to 264.5 ± 8.43 ngL^−1^ in 51% of the samples. The median concentrations of tetracycline antibiotics, which are usually applied in virology, neurology, and oncology, were low, only being measurable for oxytetracycline, in 38% of the samples in the concentration range from 3.33 ± 0.57 to 60 ± 3.39 ngL^−1^. The maximum concentration of clarithromycin (264.5 ± 8.43 ngL^−1^) measured in Sousse was far below the highest concentrations reported in Switzerland, 2.555 ngL^−1^ (Kovalova et al. 2012) and in the USA 1,420 ngL^−1^ (Oliveira et al. 2015) (Fig. 3).

We observed that the concentration of sulfamethoxazole and sulfapyridine was similar in all the HWW samples, which evidenced their usually combined therapeutic use, as reported by Batt et al. (2006). The main human metabolite of sulfapyridine, the acetylated derivative, was also frequently detected (92%). For sulfamethoxazole, the maximum concentration observed corresponded to the HWW from Sfax (494.5 ± 15.6 ngL^−1^) and was much higher than those reported by Santos et al. (2013) (307 ngL^−1^), Li and Lin (2015) (2,670 ngL^−1^), and Lien et al. (2016) (200 ngL^−1^) in HWW (Fig. 3). Trimethoprim has been the most targeted pharmaceutical in similar studies. Concentrations of up to 13000 ngL^−1^ (Thai-Hoang et al. 2016) were reported, which compared to the maximum concentration measured in the present study (175 ± 2.96 ngL^−1^ in Sfax) was 30 to 75-fold higher.

Enrofloxacin was present in more than half of the HWW at concentrations in a wide range, from 43 ± 0.7 ngL^−1^ (Sidi Bouzid) to 484.67 ± 6.45 ngL^−1^ (Monastir). In contrast, enrofloxacin was not detected in any of the effluent samples collected from hospitals in Italy (Verlicchi et al. 2012) (Fig. 3).

The anti-inflammatory ketoprofen was measured only in two hospitals, Sfax and Mahdia at 673.3 ± 25.17 ngL^−1^ and 1190 ± 24.02 ngL^−1^, respectively, which was almost two-fold as reported by Oliveira et al. (2015) 640 ngL^−1^. Mefenamic acid was also found very frequently (91%), at the highest concentrations in Tunis (4465 ± 21 ngL^−1^) and Sfax (1129.33 ± 37.9 ngL^−1^). The beta-blocker atenolol was almost ubiquitous (93%) and at very high concentrations between 2155 ± 68.11 ngL^−1^ (Sousse) and 19883.3 ± 75.4 ngL^−1^ (in Sidi Bouzid). The antiepileptic carbamazepine was found in all the samples, from 63.65 ± 9.4 ngL^−1^(Gafsa) to 562 ± 9.94 ngL^−1^ (Sousse). Its epoxy-metabolite was also present but less frequently detected (29%) and at lower concentrations (maximum of 36.5 ± 1.1 ngL^−1^ in Mahdia). These results are similar to the recently reported concentrations of carbamazepine, ciprofloxacin, ofloxacin, ketoprofen, acetaminophen, and sulfamethoxazole in Vietnam wastewater (Bui et al. 2021).

### Ecotoxicity of the hospital effluents

#### *Daphnia magna* assay

In *D. magna*’s immobilization tests, the mean EC50 values calculated after 24 and 48 h exposure to different proportions of HWW ranged from 27.6 to 79.8% (Table 3). After 48 h of exposure, the mean EC50 values obtained were between 27.6 (Tunis) and 55.3% (Sidi Bouzid). The ecotoxicity of each HWW was estimated using the classification described by Persoone et al. (2003) as shown in Table 3. According to the values, all of them belonged to class III; they were toxic. As expected, the toxicity increased with exposure time; after 48 h, the number of immobile individuals approximately doubled that measured after 24 h.

**Table 3:** Mean EC50 (%), equivalent toxic units (TU), and corresponding Persoone classification for each Tunisian hospital effluent tested on *Daphnia magna* after 24 and 48 h. Bioassay endpoint, immobility.

Hospital location	EC_50_ (%)			Persoone classification
	24 h	48 h	TU	
Monastir	67.4	31.7	1.48–3.15	Toxic effluent
Tunis	44.3	27.6	2.25–3.70	Toxic effluent
Sousse	57.6	30.6	1.75–3.33	Toxic effluent
Sfax	67.5	29.5	1.48–3.38	Toxic effluent
Gafsa	70.6	46.6	1.41–2.50	Toxic effluent
Sidi Bouzid	79.8	55.3	1.25–1.80	Toxic effluent
Mahdia	61.1	32.2	1.63–3.10	Toxic effluent

#### *Vibrio fischeri* assay

In *V. fischeri*’s bioluminescence test, the EC50 values calculated after 15- and 30-min exposure to the different proportions of HWW were in the range 5.5–17.9%, as listed in Table 4. There was little change in toxicity between 15- and 30-min exposure time. Besides, the toxicity for each HWW had maximal levels (from 18 to 13) for the effluents from the Tunis hospital. The estimated TU showed that most HWW belonged to class III, i.e., toxic. In particular, the HWW from Tunis and Sousse was the most toxic for *V. fischeri*. This hospital has the maximum number of beds (1049) and the highest total load of PPCPs (1347845.7 μgL^−1^), while the Sousse Hospital has more than half the beds (670) and a total load of PPCPs of 585714.6 μgL^−1^, the fourth part. Despite these notorious differences, the fact that both are very toxic to *V. fischeri* could be related to the presence of carbamazepine, whose median concentrations are the two highest in the HWW, 417.5 ngL^−1^ in Tunis and 562 ngL^−1^ in Sousse (Chen et al. 2019).

**Table 4:** Mean EC50 (%), toxic unit (TU) values, and Persoone classification for each Tunisian hospital tested toward *Vibrio fischeri* after 15- and 30-min exposure time.

Hospital	EC_50_ (%)			Persoone classification
	15 min	30 min	TU	
Monastir	9.47	10.47	11–10	Toxic effluent
Tunis	5.50	7.42	18–13	Toxic effluent
Sousse	7.53	9.24	14–11	Toxic effluent
Sfax	10.62	11.02	10–9	Toxic effluent
Gafsa	12.31	13.73	8–7	Toxic effluent
Sidi Bouzid	15.57	17.9	6–5.5	Toxic effluent
Mahdia	8.17	9.22	12–11	Toxic effluent

### Environmental risk assessment

The toxicity data estimated through the bioassays were correlated to some of the factor characteristics of the hospital; between toxicity and number of beds (0.460) (Table 5). Correlations found were moderate between toxicity and number of consultations (*r* = 0.460) and low for total load of pharmaceuticals (*r* = 0.264) and number of emergency cases registered in the hospital (*r* = 0.250). Unexpectedly, the number of beds appears to have a very low influence on the toxicity of the HWW (0.110).

**Table 5:** Pearson’s correlation coefficient between toxicity and hospital characteristic factors.

	Factors			
	Consultations	Emergency cases	∑Pharmaceuticals	No. of beds
Toxicity	0.460**	0.250**	0.264*	0.110*

**Highly significant differences (λ = 0.01); *significant differences (λ = 0.05)

To identify the compounds providing the highest environmental risk, HQs were calculated for each HWW sample. For pharmaceuticals, HQs for *D. magna* were in the range of 0.4 (sulfabenzamide in Sfax) – 121 (enrofloxacin in Monastir), and for *V. fischeri*, somewhat higher values were obtained, between 0.37 (sulfabenzamide in Sfax) and 234 (marbofloxacin in Mahdia). Only four substances posed no risk (HQ < 1) when present, i.e., flumequine in Monastir (0.8 for both organisms), sulfabenzamide in Sfax (0.37 for both organisms), and trimethoprim also in Sfax (0.6 for *V. fischeri*).

Concerning the toxicity of the HWW effluents, all of them were toxic to both organisms, with cumulative HQs pretty similar, for *D. magna* in the range 60.9–2.367 and *V. fischeri* 62–483 in Tunis and Mahdia, respectively. The ecotoxic effect is most likely related to the intrinsic biological activity of these compounds, which are also effective in non-target organisms.

Regarding personal care products, HQs for *D. magna* were in the range of 0.1 (4DHB in Monastir and Tunis) – 82.2 (BZT in Mahdia), and for *V. fischeri* varied between 1.1 (4DHB in Sfax) and 83.7 (BZT in Mahdia), somewhat lower as compared to pharmaceuticals, especially antibiotics. For both microorganisms, the benzotriazole BZT was, by far, the most hazardous compound with HQ > 80.

Considering the toxicity of the HWW, all of them were toxic with cumulative HQs from 5.7 (Tunis) to 101.7 (Mahdia) when *D. magna* was exposed. Similarly, when the *V. fischeri* was tested, the Tunis HWW resulted in a cumulative HQ of 5.8, whereas for the wastewater from Mahdia Hospital, the HQ was 104.5. Despite these concerning outcomes, as no organisms are directly exposed to the HWW but to the diluted effluent once in the environment, these estimates of toxicity must be seen as the worst-case scenario.

## Discussion

In the context of detecting UV filters in the analyzed hospital waters, it is noteworthy to highlight the presence of BP2. This compound is recognized for its tendency to accumulate in aquatic organisms due to its structural symmetry and relatively small size, enabling it to permeate tissues (Díaz-Cruz et al. 2019). The implications of BP2 exposure are considerable, as evidenced by its association with notable alterations in the thyroid axis in both rats and humans (Schmutzler et al. 2007), along with its correlation to endometriosis in women (Kunisue et al. 2012). Particularly high were the median concentrations of 4HB, one of the major metabolites of benzophenone-type UV. This result is concerning, as 4HB is known to induce critical DNA damage, leading to significant genotoxic effects, even more severe than those of the parent compound (Jeon 2017). This finding highlights the importance of considering the potential impacts of metabolites rather than limiting assessments solely to the parent compounds in studies of contaminants in hospital wastewater.

Beyond the scope of this investigation and for future studies, the publication “State of the climate in Europe in 2022,” jointly produced by the World Meteorological Organization (WMO) and the Copernicus Climate Change Service (C3S) of the European Union, reveals that surface solar radiation in 2022 reached the highest levels since records began in 1983, surpassing the average for the period from 1991 to 2020 by 4.9%. Notably, the UV index in Tunisia exceeds 10, indicating a very high risk of skin damage from the sun’s UV radiation which will likely lead to an increase in products with UV protection.

The elevated concentrations of benzotriazoles observed in our study can be attributed to the widespread consumption of triazole derivatives, playing pivotal roles in medicinal chemistry (Ren et al. 2014). Notably, BZT and its derivatives find diverse applications, including serving as UV filters and UV photo stabilizers in plastics, rubbers, and synthetic fibers (Maisuradze et al. 2013). Furthermore, certain BZT derivatives exhibit potent anticancer properties, exemplified by the antineoplastic agenst vorozole (VOR) and 4,5,6,7-tetrabromobenzotriazole (TBB). The imperative to develop novel anticancer drugs based on benzotriazole stems is motivated by the capacity of various benzotriazole derivatives, along with metal complexes containing benzotriazole, to overcome the side effects and limitations associated with currently employed clinical anticancer drugs (Entezar et al. 2014).

However, the extensive clinical application of BZT in medicinal chemistry extends beyond anticancer drugs to encompass antibacterial, antifungal, antiviral, antitubercular, antihypertensive, antioxidative, and anthelmintic agents (Ren et al. 2014). The observed high frequency and concentration levels of BZT in aquatic environments likely result from the amalgamation of its diverse applications. Yet, in the specific context of hospital wastewater in our investigation, the primary contribution appears to be linked to the clinical application of drugs containing BZT derivatives. These derivatives undergo metabolism after administration, break down, and eventually release the BZT moiety into the environment.

In recent years, several authors have documented the presence of BZT and its derivatives in environmental samples (Mizukawa et al. 2017; Fenni et al. 2022). Nevertheless, previous studies consistently linked their occurrence to applications in the materials industry, such as corrosion inhibition, photoionization, and UV blocking agent. To the best of the author’s knowledge, this study marks the first instance in which BZT residues are directly associated with clinical drugs. This significant discovery underscores that untreated wastewater from hospitals constitutes a notable and concerning point source of BZT contamination in the environment. These effluents are typically combined with urban wastewater and processed in wastewater treatment plants (WWTPs), where they are not efficiently eliminated (Molins-Delgado et al. 2014), ultimately being discharged into the aquatic environment. Once released, BZTs persist due to their high solubility and poor biodegradability, exhibiting half-lives of several days (Liu et al. 2012).

Regarding pharmaceuticals, the limited and occasional detection of certain extensively prescribed antibiotics, such as cephalosporins and penicillins, in this study could be attributed to the chemical instability of the common β-lactam nucleus and their short half-life in aqueous solutions (Randolph and Joseph 2018). As a result, we postulate that these pharmaceuticals might be utilized in minimal doses and/or undergo degradation during their transit into sewage discharge (Ebele et al. 2017). Additionally, various physicochemical characteristics of antibiotics can influence their fate and removal. The partition coefficient (log Kow) and solubility of antibiotics (Table 6S) dictate their adsorption affinity onto sludge or suspended particles, primarily organic components of materials. In general, compounds with high water solubility and low log Kow exhibit low adsorption capacity, whereas those with low water solubility and high log Kow demonstrate high adsorption affinity (Daghrir and Drogui 2013).

The findings for ofloxacin, nalidixic acid, and enrofloxacin align with prior studies in hospitals, indicating quinolones as the most extensively utilized class of antimicrobials in both outpatient and inpatient treatments (Kim and Hooper 2014). The notable concentration and frequent detection of these compounds can be attributed to their extensive medical usage, with quinolones and fluoroquinolones being prominently administered in hospitals for the treatment of diverse bacterial infections (Kim and Hooper 2014). Quinolones, in general, are regarded as first-line treatments for respiratory and urinary tract infections, sexually transmitted diseases, and skin structure infections (Kim and Hooper 2014). Similarly, fluoroquinolones enjoy widespread use in hospitals due to their effectiveness against hospital-acquired infections, demonstrating potent activity against various pathogenic bacteria and nosocomial infections (Polk et al. 2004).

Trimethoprim emerges as the most frequently studied antibiotic in hospital effluents and is often found in association with other antibiotics, such as sulfonamides. In our study, both trimethoprim and several sulfonamides were detected. It is noteworthy that trimethoprim and sulfamethoxazole are included in the Watch List of substances for monitoring purposes at the European Union level in the field of water policy, aligning with the EU Strategic Approach to Pharmaceuticals in the Environment (COM 2019) and the European One Health Action Plan against Antimicrobial Resistance (AMR). This inclusion supports the use of the Watch List to gain insight into the occurrence and spread of antimicrobials in the environment.

The concentrations of pharmaceuticals documented in this study closely resemble those previously recorded in northeastern Tunisia by Moslah et al. (2018), encompassing caffeine, paracetamol, propranolol, atenolol, and carbamazepine across seven WWTP effluents. According to Moslah et al. (2018), the pharmaceutical levels (expressed in sales units) in Tunisia, referencing data from the Tunisian Ministry of Public Health, followed this order: analgesics and anti-inflammatories (up to 21634811) > antibiotics (up to 6,055.895) > cortico-adrenal hormones (up to 3086347) > psychiatric drugs (up to 1254015) > histamine H1 and H2 receptor antagonists (up to 800455) > ß-blockers (up to 767410). Notably, our study’s results underscore coherence between pharmaceutical prescriptions in Tunisia and the concentrations detected for most target compounds in HWW, potentially elucidating the elevated concentrations observed.

Regarding the fluctuations in pharmaceutical concentrations in the analyzed HWW, earlier studies have also indicated substantial variations attributed to diverse factors, including the hospital’s nature and specialties, bed count, room and service types, prescription patterns, and the number and clinical situations of hospitalized patients (inpatients or outpatients). Additional considerations encompass the average flow rate of wastewater effluent and sampling methodologies (Oliveira et al. 2015; Carraro et al. 2016).

Hospital wastewater comprises a complex mixture of numerous substances that may interact collectively in an additive, synergistic, or antagonistic manner, potentially causing toxic effects in organisms. Prior research has indicated that the concurrent presence of pharmaceutical residues in HWW, known as the cocktail effect, can lead to adverse impacts on aquatic organisms (Ben Mansour et al. 2007; Eguchi 2004). The heightened toxicity observed in this study is likely exacerbated by the broad spectrum of pharmaceuticals administered in a hospital setting, suggesting the occurrence of synergistic actions.

While a presumed relationship between toxicity and pharmaceutical concentration exists, it is noteworthy that the toxicity of HWW might also be influenced by the number of consultations and medical emergencies. In routine consultations and hospital emergencies, a substantial number of diagnostic and analytical tests are conducted, and direct care is provided to alleviate symptoms through the administration of antipyretics, anti-inflammatories, antibiotics, β-blockers, anxiolytics, and other pharmaceuticals. Additionally, due to the overcrowding of hospital emergency services, a common occurrence in developed countries, patients may remain in the hospital for several hours before being discharged. These factors collectively contribute to the complexity of the HWW composition and the potential for increased toxicity.

Given that our analysis involved real samples rather than a laboratory-controlled exposure of standard mixtures, the observed toxicity could be partially attributed to the potential presence of toxic compounds resulting from the use of hypochlorite and iodized substances in some hospitals for the disinfection of liquid discharges. Sodium hypochlorite is commonly employed to disinfect water and prevent the spread of pathogenic microorganisms. Several authors have reported that some toxic compounds, such as chloramines, can form after treatment with chlorine (Herrera 2013). While the hospitals in our study did not explicitly treat the wastewater stream, we cannot dismiss the possibility that they might sporadically disinfect certain departments or waste discharge systems during our sampling periods. Consequently, the observed toxicity might be partly explained by chlorine disinfectants in the wastewater, reacting with organic matter and generating halogenated organic compounds (AOX), which are known to be toxic to aquatic organisms (Emmanuel et al. 2004). It is worth noting that the physicochemical characteristics of the HWW samples in our study showed concentrations of up to 0.5 mgL^-^^1^ AOX (halogenated organic compounds) (Table 3S).

Recent findings by Carraro et al. (2016) indicated that HWW had ammonium ion concentrations ranging from 9 to 42 mgL^-1^, surpassing levels observed in municipal wastewater in the same area (not detected—28.2 mgL^-1^). The elevated ammonium concentration was suggested to be linked to the degradation of amine compounds present in the water, including those from pharmaceuticals (Lin et al. 2008). Previous research by Liu et al. (2011) demonstrated the ecotoxic effects on the alga Selenastrum capricornutum caused by the antibiotics erythromycin, ciprofloxacin, and sulfamethoxazole, inhibiting electron transport, photophosphorylation, and carbon uptake. Comparing these results with our study, the observed mortality may be attributed to the extensive use of antibiotics in hospitals, particularly those identified in our study, such as ofloxacin and sulfapyridine.

Several studies have affirmed the toxicity of numerous antibiotics, such as trimethoprim, clarithromycin, and ofloxacin, to aquatic organisms. For instance, Mendoza et al. (2015) reported HQs greater than 10 for trimethoprim, clarithromycin, and ofloxacin in a hospital located in Valencia, Spain. Additionally, Santos et al. (2013) suggested the inclusion of six antibiotics—ciprofloxacin, ofloxacin, sulfamethoxazole, azithromycin, clarithromycin, and metronidazole—on a list of 11 pharmaceuticals identified as potentially hazardous for aquatic organisms, recommending their inclusion in future monitoring programs.

For these reasons, wastewater management is a critical component of hospital infrastructure, ensuring environmental safety and public health.

## Conclusions

After a 3-month sampling period, we determined the concentration of pharmaceuticals and personal care products released by Tunisian hospitals to the environment. Chemical analysis by HPLC-MS/MS revealed the presence of a variety of antibiotics such as enrofloxacin, marbofloxacin, oxytetracycline, pipemidic acid, sulfamethoxazole, acetaminophen, and mefenamic acid, along with other classes of pharmaceuticlas, including atenolol, carbamazepine, and the stimulant caffeine. Expectedly, anti-inflammatories, quinolones, fluoroquinolones, and sulfonamides were the dominant therapeutic groups. Personal care products were also found including the UV filters benzophenone-3, benzophenone-2, and the metabolites benzophenone 1, 4HB and 4DHB, and UV blockers, such as BZTs. To the best of the authors’ knowledge, UVF and BZT were investigated for the first time in wastewater effluents from Tunisian hospitals, due to the high UV index reached in the country, with values of 10 or eventually higher, and thus the concentrations found can serve as background levels in hospital wastewater discharges. The risk assessment performed suggests that these compounds could pose a high risk for the tested organisms, *Daphnia magna* and *Vibrio fischeri*, if they were directly exposed to HWWs. According to our results, the development and application of wastewater management strategies in hospitals as well as the improvement of WWTP technologies are necessary to reduce the load of PPCPs being of particular concern antibiotics due to the potential spread of bacteria resistant to them. Especially surprising was the high load of BZT found in the HWW, explained by its extensive clinical application associated with new-generation medicines as anticancer drugs but also antibacterial, antifungal, antiviral, antitubercular, antihypertensive, antioxidative, and anthelmintic agents. Consequently, appropriate regulations might be developed based on environmental monitoring and ecotoxicity data.

### Supplementary information

ESM 1 (DOCX 83 kb)

## Data Availability

We provide a file with Supporting Data.
