# Supplementary material for: Pharmaceuticals and personal care products in Tunisian hospital wastewater: occurrence and environmental risk
Source: Environ Sci Pollut Res Int. 2023 Dec 8;31(2):2716–31. doi: 10.1007/s11356-023-31220-1 (PMC10791778; doi:10.1007/s11356-023-31220-1)
Supplement: Supplementary file 1 — (DOCX 83 kb) [file 11356_2023_31220_MOESM1_ESM.docx]

**Supplementary Information**

OCCURRENCE AND FATE OF PHARMACEUTICALS AND PERSONAL CARE PRODUCTS IN THE TUNISIAN HOSPITAL WASTEWATERS

Emna Nasri^1, 2^, Ana C. Soler^3^, Carlos Barata^3^, Hedi ben Mansour^1^, M. Silvia Díaz-Cruz^3^*

^1^ *Research Unit of Analysis and Process Applied to the Environmental e APAE Higher Institute of Applied Sciences and Technology Mahdia, University of Monastir, Monastir*

^2^ *Laboratory of Biotechnology and Bio-Monitoring of the Environment and Oasis Ecosystems,Department of Life Sciences, Faculty of Sciences of Gafsa, Sidi Ahmed Zarroug University Campus,Gafsa 2112, Tunisia*

^3^*Department of Environmental Chemistry, Institute of Environmental Assessment and Water Research (IDAEA), Spanish Council for Scientific Research (CSIC), Jordi Girona 18-26, E-08034 Barcelona, Spain*

Table 1S. MS/MS operation conditions for the ion transitions monitored in the analysis of the selected PPCPs. In all cases the precursor ion was [M+H]^+^. DP, decluttering potential, C.E., collision energy, CxP, collision cell exit potential.

| **Compound (acronym)** | **1st. Transition** | **DP (V)** | **CE (eV)** | **CxP (eV)** | **2nd. Transición** | **DP (V)** | **CE (eV)** | **CxP (eV)** | **Internal Standard** |  |
| --- | --- | --- | --- | --- | --- | --- | --- | --- | --- | --- |
| **Benzophenone 3 (BP3)** | 229>151 | 40 | 25 | 12 | 229>105 | 40 | 27 | 16 | BP3-d_5_ |  |
| **Benzophenone 1 (BP1)** | 215>137 | 40 | 27 | 10 | 215>105 | 40 | 29 | 6 | BP3-d_5_ |  |
| **Benzophenone 2 (BP2)** | 247>137 | 46 | 25 | 8 | 247>109 | 46 | 45 | 8 | BP3-d_5_ |  |
| **4-hydroxybenzophenone (4HB)** | 199>121 | 40 | 25 | 8 | 199>105 | 40 | 27 | 8 | BP3-d_5_ |  |
| **4,4'-dihydroxybenzophenone**  **(4DHB)** | 215>121 | 45 | 27 | 8 | 215>93 | 45 | 45 | 6 | BP3-d_5_ |  |
| **2,2'-dihydroxy-4-methoxybenzophenone (DHMB)** | 245>121 | 43 | 29 | 8 | 245>151 | 43 | 27 | 12 | BP3-d_5_ |  |
| **3-(4-Methylbenzylidene)camphor (4-MBC)** | 255>105 | 61 | 45 | 6 | 255>212 | 61 | 29 | 14 | 4MBC-d_4_ |  |
| **Ethyl-4-*p*-aminobenzoic acid (EtPABA)** | 166>138 | 41 | 20 | 10 | 166>120 | 41 | 25 | 28 | 4MBC-d_4_ |  |
| **Ethylhexyl dimethyl-4-*p*-aminobenzoic acid (ODPABA)** | 278>166 | 86 | 43 | 42 | 278>151 | 86 | 27 | 40 | 4MBC-d_4_ |  |
| **1H-Benzotriazol (BZT)** | 120>65 | 56 | 31 | 4 | 120>92 | 56 | 25 | 16 | BZT-d_4_ |  |
| **5-Methyl-(1H-Benzotriazol ) (MeBZT)** | 134>79 | 46 | 29 | 10 | 134>95 | 46 | 35 | 14 | BZT-d_4_ |  |
| **5,6-Dimethyl-1H-benzotriazole (DMBZT)** | 148>77 | 56 | 39 | 2 | 148>93 | 56 | 27 | 6 | BZT-d_4_ |  |
| **2-(2H-Benzotriazol-2-yl)-p-cresol (UVP)** | 226>120 | 46 | 25 | 10 | 226>107 | 46 | 19 | 18 | BZT-d_4_ |  |
| **Clarithromycin (CLR)** | 749>158 | 76 | 39 | 10 | 749>83 | 76 | 84 | 5 | Clarithromycin-N-methyl-d_3_ |  |
| **Enoxacin (ENO)** | 321 > 234 | 56 | 10 | 31 | 321 > 257 | 56 | 16 | 10 | Clarithromycin-N-methyl-d_3_ |  |
| **Enrofloxacin (ENR)** | 360 > 316 | 86 | 29 | 16 | 360 > 245 | 10 | 35 | 18 | Erithromycin-N,N-dimethyl-^13^C | - |
| **Flumequine (FLU)** | 262>244 | 51 | 29 | 14 | 262>202 | 51 | 45 | 10 | Flumequine-^13^C |  |
| **Marbofloxacin (MAR)** | 363 > 72 | 81 | 10 | 53 | 363 > 320 | 20 | 81 | 10 | Flumequine-^13^C |  |
| **Nalidixic acid (NAX)** | 233>215 | 61 | 19 | 14 | 233>187 | 61 | 37 | 12 | Flumequine-^13^C |  |
| **Oxolinic acid (OXO)** | 262>244 | 36 | 25 | 12 | 262>216 | 36 | 41 | 16 | Flumequine-^13^C |  |
| **Oxytetracycline (OTC)** | 461>426 | 61 | 27 | 10 | 461>443 | 61 | 19 | 14 | Trimethoprim-d_3_ |  |
| **Succynil-Sulfathiazole (SST)** | 356>256 | 71 | 25 | 16 | 356>192 | 71 | 33 | 16 | Sulfamethazine-d_4_ |  |
| **Sulfadiazine (SDZ)** | 251>156 | 46 | 27 | 10 | 251>108 | 46 | 30 | 8 | Sulfamethazine-d_4_ |  |
| **N^4^-acetylsulfadiazidine (acSDZ)** | 293>134 | 65 | 30 | 12 | 293>198 | 65 | 30 | 12 | Sulfamethazine-d_4_ |  |
| **Sulfamerazine (SMR)** | 265>156 | 61 | 27 | 8 | 265>92 | 61 | 47 | 6 | Sulfamethazine-d_4_ |  |
| **N^4^-acetylsulfamerazine (acSMR)** | 307>134 | 60 | 35 | 8 | 307>110 | 60 | 35 | 8 | Sulfamethazine-d_4_ |  |
| **N^4^-acetylsulfamethazine (acSMZ)** | 321>134 | 86 | 35 | 4 | 321>124 | 86 | 35 | 4 | Sulfamethazine-d_4_ |  |
| **Sulfamethoxazole (SMX)** | 254>108 | 56 | 27 | 10 | 254>156 | 56 | 25 | 10 | Sulfamethazine-d_4_ |  |
| **N^4^-acetylsulfamethoxazole (acSMX)** | 296>198 | 60 | 30 | 10 | 296>134 | 60 | 30 | 10 | Sulfamethazine-d_4_ |  |
| **Sulfamethoxypyridazine (SMP)** | 281>126 | 66 | 27 | 12 | 281>156 | 66 | 27 | 14 | Sulfamethazine-d_4_ |  |
| **Sulfapyridine (SPY)** | 250>156 | 51 | 28 | 12 | 250>92 | 51 | 31 | 6 | Sulfamethazine-d_4_ |  |
| **N^4^-acetylsulfapyridine (acSPY)** | 292>134 | 70 | 30 | 8 | 292>198 | 70 | 30 | 8 | Sulfamethazine-d_4_ |  |
| **Sulfaquinoxaline (SQX)** | 301>156 | 76 | 25 | 10 | 301>92 | 76 | 47 | 12 | Sulfamethazine-d_4_ |  |
| **Sulfathiazole (STZ)** | 256>156 | 40 | 25 | 14 | 256>92 | 40 | 25 | 10 | Sulfamethazine-d_4_ |  |
| **Sulfisomidin (SSM)** | 279>124 | 76 | 33 | 8 | 279>186 | 76 | 23 | 14 | Sulfamethazine-d_4_ |  |
| **Sulfadimethoxine (SDM)** | 311>156 | 76 | 31 | 8 | 311>92 | 76 | 31 | 6 | Sulfamethazine-d_4_ |  |
| **Sulfanitran (SNT)** | 336>156 | 66 | 17 | 12 | 336>198 | 66 | 29 | 14 | Flumequine-^13^C_3_ |  |
| **Sulfabenzamide (SBD)** | 277>156 | 56 | 17 | 10 | 277>92 | 56 | 41 | 6 | Clarithromycin-N-methyl-d_3_ |  |
| **Trimethoprim (TMP)** | 291>230 | 76 | 33 | 20 | 291>261 | 76 | 35 | 12 | Trimethoprim-d_3_ |  |
| **Penicillin V (PEN)** | 351 > 160 | 66 | 17 | 26 | 351 > 114 | 66 | 47 | 18 | Flumequine-^13^C_3_ |  |
| **Pipemidic acid (PIP)** | 304>217 | 66 | 29 | 20 | 304>189 | 66 | 45 | 16 | BZT-d_4_ |  |
| **Acetaminophen/Paracetamol (AMP)** | 152>110 | 71 | 23 | 18 | 152>65 | 71 | 45 | 10 | Trimethoprim-d_3_ |  |
| **Atenolol (ATL)** | 267>116 | 46 | 31 | 8 | 267>133 | 46 | 39 | 22 | Trimethoprim-d_3_ |  |
| **Ketoprofen (KEP)** | 255>105 | 66 | 33 | 18 | 255>209 | 66 | 25 | 10 | BP3-d_5_ |  |
| **Mefenamic acid (MFA)** | 242>224 | 46 | 23 | 22 | 242>209 | 46 | 41 | 14 | Mefenamic acid-d_3_ |  |
| **Naproxen (NAP)** | 231>185 | 56 | 17 | 14 | 231>115 | 56 | 77 | 10 | Flumequine-^13^C |  |
| **Carbamazepine (CBZ)** | 237>194 | 61 | 29 | 14 | 237>192 | 61 | 31 | 12 | Carbamazepine-d_10_ |  |
| **Carbamazepine 10,11-epoxy (CBZ-epoxy))** | 253>180 | 36 | 35 | 12 | 253>151 | 36 | 109 | 10 | Carbamazepine-d_10_ |  |
| **Ofloxacin (OFL)** | 362>318 | 56 | 29 | 18 | 362>261 | 56 | 41 | 18 | Sulfamethazine-d_4_ |  |
| **Caffeine (CAF)** | 195>109 | 56 | 31 | 8 | 195>138 | 56 | 29 | 10 | Trimethoprim-d_3_ |  |
|  |  |  |  |  |  |  |  |  |  |  |

Table 2S. LC-MS/MS method performance; calibration range, coefficient of correlation (r^2^), recovery rates in percentage (R (%)), relative standard deviation in percentage for n=6 (RSD (%)), method limit of detection (LOD), and method limit of quantification (LOQ)).

| **Compound (acronym)** | **Calibration range (ngmL^-1^)** | **r^2^** | **R (%)**  **(spike 50 ngL^-1^)** | **RSD (%)** | **LOD (ngL^-1^)** | **LOQ (ngL^-1^)** |
| --- | --- | --- | --- | --- | --- | --- |
| **Benzophenone 3 (BP3)** | 1-700 | 0.9999 | 116 | 16.8 | 0.18 | 0.60 |
| **Benzophenone 1 (BP1)** | 1-700 | 0.9994 | 70.9 | 13.8 | 0.44 | 1.46 |
| **Benzophenone 2 (BP2)** | 1-700 | 0.9996 | 79.4 | 8.0 | 0.60 | 2.03 |
| **4-hydroxybenzophenone (4HB)** | 1-700 | 0.9999 | 91.5 | 6.4 | 0.60 | 2.01 |
| **4,4'-dihydroxybenzophenone**  **(4DHB)** | 1-700 | 0.9996 | 45.8 | 19.6 | 1.18 | 3.95 |
| **2,2'-dihydroxy-4-methoxybenzophenone (DHMB)** | 1-700 | 0.9999 | 80.8 | 4.4 | 0.87 | 2.90 |
| **3-(4-Methylbenzylidene) camphor (4-MBC)** | 1-700 | 0.9999 | 118 | 18.9 | 0.44 | 1.47 |
| **Ethyl-4-*p*-aminobenzoic acid (EtPABA)** | 1-700 | 0.9995 | 90.1 | 13.2 | 0.33 | 1.08 |
| **Ethylhexyl dimethyl-4-*p*-aminobenzoic acid (ODPABA)** | 1-700 | 0.9999 | 113 | 13.0 | 0.36 | 1.19 |
| **1H-Benzotriazol (BZT)** | 1-700 | 0.9997 | 102 | 20.1 | 0.33 | 1.12 |
| **5-Methyl-(1H-benzotriazol) (MeBZT)** | 1-700 | 0.9988 | 106 | 15.9 | 0.17 | 0.59 |
| **5,6-Dimethyl-1H-benzotriazole (DMBZT)** | 1-700 | 0.9988 | 92.1 | 9.9 | 0.45 | 1.52 |
| **2-(2H-Benzotriazole-2-yl)-p-cresol (UVP)** | 1-700 | 0.9998 | 96.1 | 12.8 | 0.19 | 0.66 |
| **Clarithromycin (CLR)** | 1-700 | 0.9976 | 87.3 | 18.3 | 0.21 | 0.64 |
| **Enoxacin (ENO)** | 1-700 | 0.9982 | 89.4 | 15.2 | 0.10 | 0.32 |
| **Enrofloxacin (ENR)** | 1-700 | 0.9987 | 106 | 2.6 | 0.30 | 0.96 |
| **Flumequine (FLU)** | 1-700 | 0.9999 | 82.9 | 7.4 | 0.21 | 0.70 |
| **Marbofloxacin (MAR)** | 1-700 | 0.9980 | 74.2 | 8.6 | 0.71 | 2.43 |
| **Nalidixic acid (NAX)** | 1-700 | 0.9999 | 74.0 | 8.6 | 0.18 | 0.62 |
| **Oxolinic acid (OXO)** | 1-700 | 0.9980 | 107 | 20.0 | 0.13 | 0.45 |
| **Oxytetracycline (OTC)** | 1-700 | 0.9924 | 65.4 | 16.2 | 0.26 | 0.88 |
| **Succynil-Sulfathiazole (SST)** | 1-700 | 0.9999 | 92.5 | 8.2 | 0.11 | 0.37 |
| **Sulfadiazine (SDZ)** | 1-700 | 0.9999 | 76.4 | 2.1 | 0.31 | 1.22 |
| **N^4^-acetylsulfadiazidine (acSDZ)** | 1-700 | 0.9999 | 110 | 12.3 | 0.11 | 0.35 |
| **Sulfamerazine (SMR)** | 1-700 | 0.9999 | 96.8 | 2.1 | 1.21 | 4.0 |
| **N^4^-acetylsulfamerazine (acSMR)** | 1-700 | 0.9999 | 123 | 4.1 | 0.17 | 0. 58 |
| **N^4^-acetylsulfamethazine (acSMZ)** | 1-700 | 0.9999 | 74.3 | 3.4 | 0.14 | 0.45 |
| **Sulfamethoxazole (SMX)** | 1-700 | 0.9999 | 95.9 | 6.7 | 0.60 | 2.02 |
| **N^4^-acetylsulfamethoxazole (acSMX)** | 1-700 | 0.9995 | 122 | 7.6 | 0.35 | 1.17 |
| **Sulfamethoxypyridazine (SMP)** | 1-700 | 0.9999 | 115 | 5.4 | 0.12 | 0.40 |
| **Sulfapyridine (SPY)** | 1-700 | 0.9999 | 111 | 2.9 | 0.35 | 1.18 |
| **N^4^-acetylsulfapyridine (acSPY)** | 1-700 | 0.9999 | 90.9 | 7.6 | 0.25 | 0.85 |
| **Sulfaquinoxaline (SQX)** | 1-700 | 0.9999 | 93.4 | 5.5 | 0.29 | 0.98 |
| **Sulfathiazole (STZ)** | 1-700 | 0.9999 | 106 | 6.0 | 0.11 | 0.38 |
| **Sulfisomidin (SSM)** | 1-700 | 0.9998 | 92.7 | 7.1 | 0.15 | 0.50 |
| **Sulfadimethoxine (SDM)** | 1-700 | 0.9993 | 89.9 | 2.4 | 0.49 | 1.65 |
| **Sulfanitran (SNT)** | 1-700 | 0.9999 | 90.2 | 3.3 | 0.32 | 1.07 |
| **Sulfabenzamide (SBD)** | 1-700 | 0.9998 | 103 | 5.2 | 0.22 | 0.6 |
| **Trimethoprim (TMP)** | 1-700 | 0.9996 | 101 | 2.6 | 0.36 | 1.21 |
| **Penicillin V (PEN)** | 1-700 | 0.9981 | 62.3 | 18.6 | 0.27 | 0.91 |
| **Pipemidic acid (PIP)** | 1-700 | 0.9998 | 82.3 | 7.2 | 0.60 | 2.0 |
| **Acetaminophen/Paracetamol (AMP)** | 1-700 | 0.9999 | 97.3 | 2.1 | 0.19 | 0.63 |
| **Atenolol (ATL)** | 1-700 | 0.9908 | 89.2 | 9.3 | 0.39 | 1.32 |
| **Ketoprofen (KEP)** | 1-700 | 0.9999 | 127 | 7.2 | 0.27 | 0.90 |
| **Mefenamic acid (MFA)** | 1-700 | 0.9996 | 105 | 10.5 | 0.31 | 1.03 |
| **Naproxen (NAP)** | 1-700 | 0.9999 | 110 | 7.5 | 0.77 | 2.56 |
| **Carbamazepine (CBZ)** | 1-700 | 0.9998 | 106 | 9.4 | 0.28 | 0.94 |
| **Carbamazepine 10,11-epoxy (CBZ-epoxy)** | 1-700 | 0.9998 | 83.7 | 6.8 | 0.19 | 0.66 |
| **Ofloxacin (OFL)** | 1-700 | 0.9999 | 72.5 | 7.1 | 0.86 | 2.89 |
| **Caffeine (CAF)** | 1-700 | 0.9999 | 101 | 9.1 | 1.14 | 3.80 |
|  |  |  |  |  |  |  |

Table 3S. Physicochemical data of the hospital wastewater samples.

| **Hospital** | **BOD**  **(mgL^-1^)** | | **COD**  **(mgL^-1^)** | **TSS**  **(mgL^-1^)** | **TOC**  **(mgL^-1^)** | **NO_3_**  **(mgL^-1^)** | **AOX**  **(mgL^-1^)** | **Turbidity**  **(NTU)** | **Conductivity**  **(μscm^-1^)** | **pH** | **DCO/BOD** | **MO** | **Hg**  **(mgL^-1^)** | **Cd**  **(mgL^-1^)** | **Cu**  **(mgL^-1^)** | **Ni**  **(mgL^-1^)** | **Pb**  **(mgL^-1^)** |
| --- | --- | --- | --- | --- | --- | --- | --- | --- | --- | --- | --- | --- | --- | --- | --- | --- | --- |
|  |  |  | |  |  |  |  |  |  |  |  |  |  |  |  |  |  |
| **HW Monastir** | 747 | 1580 | | 805 | 562 | < 0.5 | < 0.5 | 481 | 5.51 | 7.06 | 2.11 | 1024.6 | 0.019 | 0.029 | 0.068 | 0.024 | 0.077 |
| **HW Sousse** | 555 | 1720 | | 1175 | 405 | < 0.5 | < 0.5 | 330 | 5.15 | 7.67 | 3 | 943.33 | 0.0073 | 0.035 | 0.059 | 0.027 | 0.063 |
| **HW Gafsa** | 472 | 1520 | | 1085 | 356 | < 0.5 | < 0.5 | 100 | 3.31 | 7.62 | 3.2 | 821.33 | 0.0024 | 0.028 | 0.035 | 0.021 | 0.063 |
| **HW Mahdia** | 385 | 1560 | | 1155 | 256 | < 0.5 | < 0.5 | 113.6 | 3.69 | 7.69 | 4.05 | 776.66 | 0.0028 | 0.028 | 0.060 | 0.025 | 0.045 |
| **HW Sfax** | 433 | 1300 | | 890 | 332 | < 0.5 | < 0.5 | 389 | 4.82 | 7.35 | 3 | 722 | 0.0087 | 0.027 | 0.067 | 0.036 | 0.045 |
| **HW Tunis** | 640 | 1660 | | 1450 | 495 | < 0.5 | < 0.5 | 149 | 7.14 | 7.35 | 2.5 | 980 | 0.0186 | 0.038 | 0.074 | 0.036 | 0.071 |
| **HW S.Bouzid** | 430 | 860 | | 1060 | 340 | < 0.5 | < 0.5 | 104 | 4.21 | 7.63 | 2 | 573.33 | 0.0079 | 0.021 | 0.027 | 0.010 | 0.018 |

BOD: biochemical oxygen demand; COD: chemical oxygen demand; TSS: total suspended solids; TOC: total organic carbon; NO_3_: nitrates; AOX: absorbable organically bound halogens; COD/BOD: ratio of the biodegradability; MO: Organic materials; HW Monastir: hospital wastewater of Fattouma Bourgiba (Monastir); HW Sousse: hospital wastewater of Farhat Hached (Sousse); HW Gafsa: hospital wastewater of Houcine Bouzaiene (Gafsa); HW Mahdia: hospital wastewater of Taher Sfar (Mahdia); HW Sfax : hospital wastewater of Hedi Chaker (Sfax); HW Tunis: hospital water of Charles Nicolle (Tunis) ; HW S.Bouzid: hospital wastewater of Sidi Bouzid

Table 4S: Concentrations of the target PPCPs in the selected hospitals’ wastewaters. 1: February 2019, 2: March, 2019, and 3: April 2019.

n.d.: not detected. <LOQ detected but below the limit of quantification.

| **Conc.**  **(ngL^-1^)** | **BP3** | **BP1** | **4HB** | **4DHB** | **BP2** | **BZT** | **UVP** | **DMBZT** | **MeBZT** |
| --- | --- | --- | --- | --- | --- | --- | --- | --- | --- |
| **Gafsa1** | 16,2 | n.d. | n.d. | n.d. | n.d. | 71,7 | n.d. | n.d. | n.d. |
| **Gafsa2** | 32,4 | n.d. | 335 | n.d. | n.d. | 34,6 | n.d. | n.d. | n.d. |
| **Gafsa3** | 23,9 | n.d. | 107 | n.d. | n.d. | 48,7 | n.d. | n.d. | n.d. |
| **Monastir1** | n.d. | n.d. | n.d. | n.d. | n.d. | n.d. | n.d. | n.d. | n.d. |
| **Monastir2** | n.d. | n.d. | n.d. | 12 | n.d. | 261 | n.d. | n.d. | n.d. |
| **Monastir3** | n.d. | n.d. | n.d. | n.d. | 154 | 247 | n.d. | n.d. | n.d. |
| **Sousse1** | n.d. | n.d. | n.d. | n.d. | 43 | 210,75 | n.d. | 374 | n.d. |
| **Sousse2** | 39,6 | n.d. | n.d. | n.d. | n.d. | 128,85 | 20 | 229 | n.d. |
| **Sousse3** | 35,1 | n.d. | n.d. | n.d. | n.d. | 169,8 | 3 | 10 | n.d. |
| **S.Bouzid1** | 61,7 | n.d. | n.d. | n.d. | n.d. | n.d. | n.d. | 48 | n.d. |
| **S.Bouzid2** | 53 | n.d. | n.d. | n.d. | 22 | 87,8 | <LOQ | n.d. | n.d. |
| **S.Bouzid3** | 42,4 | 35 | 195 | n.d. | n.d. | 69,2 | n.d. | n.d. | n.d. |
| **Sfax1** | 2,99 | n.d. | 73 | n.d. | n.d. | 233 | n.d. | n.d. | 183 |
| **Sfax2** | 2,87 | n.d. | n.d. | n.d. | 31 | 139 | 52 | n.d. | n.d. |
| **Sfax3** | 1,43 | n.d. | n.d. | n.d. | n.d. | 273 | n.d. | n.d. | 192 |
| **Tunis1** | 2,18 | n.d. | n.d. | n.d. | n.d. | 118 | n.d. | n.d. | n.d. |
| **Tunis2** | n.d. | n.d. | n.d. | 13 | 28 | n.d. | n.d. | n.d. | n.d. |
| **Tunis3** | 7,3 | n.d. | n.d. | n.d. | n.d. | 236 | n.d. | n.d. | n.d. |
| **Mahdia1** | 118 | n.d. | n.d. | 107 | 83 | n.d. | n.d. | n.d. | n.d. |
| **Mahdia2** | 34,1 | n.d. | n.d. | n.d. | n.d. | 121 | 14 | n.d. | n.d. |
| **Mahdia3** | 40,05 | n.d. | n.d. | n.d. | 22 | 242 | n.d. | n.d. | n.d. |

|  | |  |  | |  | |  | |  | |  | |  | |  | |  | |  | |
| --- | --- | --- | --- | --- | --- | --- | --- | --- | --- | --- | --- | --- | --- | --- | --- | --- | --- | --- | --- | --- |
|  | |  |  | |  | |  | |  | |  | |  | |  | |  | |  | |
| **Conc.**  **(ngL^-1^)** | |  | **Ofloxacin** | | **SDZ** | | **SMX** | | **SPY** | | **acSPY** | | **SDM** | | **Sulfabenzamide** | | **Sulfadimethoxine** | | **Sulfamerazine** | |
| **Gafsa1** | |  | 22400 | | n.d. | | n.d. | | 469 | | n.d. | | n.d. | | n.d. | | n.d. | | n.d. | |
| **Gafsa2** | |  | 15600 | | n.d. | | n.d. | | 2210 | | n.d. | | n.d. | | n.d. | | n.d. | | n.d. | |
| **Gafsa3** | |  | 18400 | | n.d. | | n.d. | | 1348,9 | | n.d. | | n.d. | | n.d. | | n.d. | | n.d. | |
| **Monastir1** | |  | 46700 | | n.d. | | 1127 | | 269 | | n.d. | | n.d. | | n.d. | | n.d. | | n.d. | |
| **Monastir2** | |  | 49400 | | n.d. | | 1550 | | 250 | | n.d. | | n.d. | | n.d. | | n.d. | | 160 | |
| **Monastir3** | |  | 33050 | | n.d. | | 1338,5 | | 244,5 | | n.d. | | n.d. | | n.d. | | n.d. | | n.d. | |
| **Sousse1** | |  | 78100 | | n.d. | | 684 | | 434,59 | | n.d. | | n.d. | | n.d. | | n.d. | | n.d. | |
| **Sousse2** | |  | 89600 | | n.d. | | 467 | | n.d. | | n.d. | | 81,3 | | n.d. | | 124 | | n.d. | |
| **Sousse3** | |  | 68850 | | n.d. | | 425,5 | | 527,41 | | n.d. | | 71,95 | | n.d. | | n.d. | | n.d. | |
| **S.Bouzid1** | |  | 20600 | | n.d. | | n.d. | | 146 | | n.d. | | n.d. | | n.d. | | n.d. | | n.d. | |
| **S.Bouzid2** | |  | 27900 | | n.d. | | n.d. | | 253 | | n.d. | | n.d. | | n.d. | | n.d. | | n.d. | |
| **S.Bouzid3** | |  | 22750 | | n.d. | | n.d. | | 246,5 | | n.d. | | n.d. | | n.d. | | n.d. | | n.d. | |
| **Sfax1** | |  | 47300 | | 110 | | 634 | | 2160 | | 254 | | n.d. | | n.d. | | n.d. | | n.d. | |
| **Sfax2** | |  | 66500 | | 66,9 | | 455 | | 1061 | | 147 | | 13,3 | | 4 | | 98 | | n.d. | |
| **Sfax3** | |  | 56900 | | 88,45 | | 364,5 | | 1310,5 | | 185,5 | | 25,95 | | n.d. | | n.d. | | n.d. | |
| **Tunis1** | |  | 37700 | | 625 | | 303 | | 10080 | | 1074 | | n.d. | | n.d. | | n.d. | | n.d. | |
| **Tunis2** | |  | 62400 | | 818 | | 496 | | 11900 | | 1310 | | n.d. | | n.d. | | n.d. | | n.d. | |
| **Tunis3** | |  | 50050 | | 571,5 | | 249,5 | | 10990 | | 1042 | | n.d. | | n.d. | | n.d. | | n.d. | |
| **Mahdia1** | |  | 36300 | | 50,1 | | 771 | | 481 | | n.d. | | n.d. | | n.d. | | n.d. | | n.d. | |
| **Mahdia2** | |  | 35300 | | n.d. | | 626 | | 438 | | n.d. | | n.d. | | n.d. | | n.d. | | n.d. | |
| **Mahdia3** | |  | 23950 | | 109,2 | | 845,5 | | 509,5 | | n.d. | | n.d. | | n.d. | | n.d. | | n.d. | |
| \|  \| \| --- \| | |  |  | |  | |  | |  | |  | |  | |  | |  | |  | |
| **Conc. (ngL^-1^)** | **Sulfamethoxazole** | | | **Sulfanitran** | | **Sulfapyridine** | | **Trimethoprim** | | **Clarithromycin** | | **Enoxacin** | | **Enrofloxacin** | | **Flumequina** | | **Marbofloxacin** | **Oxolinic acid** |  |
| **Gafsa1** | n.d. | | | n.d. | | n.d. | | 62,7 | | n.d. | | 116 | | n.d. | | 114 | | 94 | n.d. |  |
| **Gafsa2** | n.d. | | | n.d. | | 100 | | 35,7 | | n.d. | | n.d. | | n.d. | | n.d. | | n.d. | n.d. |  |
| **Gafsa3** | n.d. | | | n.d. | | n.d. | | 62,7 | | n.d. | | 135 | | n.d. | | n.d. | | n.d. | n.d. |  |
| **Monastir1** | n.d. | | | n.d. | | n.d. | | 63,8 | | n.d. | | 244 | | n.d. | | n.d. | | n.d. | n.d. |  |
| **Monastir2** | n.d. | | | n.d. | | n.d. | | 70,8 | | n.d. | | n.d. | | 244 | | 8 | | n.d. | 24 |  |
| **Monastir3** | 175 | | | n.d. | | n.d. | | 81,9 | | n.d. | | n.d. | | 1210 | | n.d. | | n.d. | n.d. |  |
| **Sousse1** | n.d. | | | n.d. | | n.d. | | 64,5 | | n.d. | | n.d. | | 205 | | n.d. | | 19 | n.d. |  |
| **Sousse2** | n.d. | | | n.d. | | 89 | | 65,8 | | 33 | | n.d. | | 178 | | n.d. | | 224 | n.d. |  |
| **Sousse3** | 461 | | | 31 | | n.d. | | 45,15 | | 460 | | n.d. | | 261 | | n.d. | | 131 | n.d. |  |
| **S.Bouzid1** | n.d. | | | n.d. | | n.d. | | n.d. | | n.d. | | n.d. | | n.d. | | 16 | | 987 | 23 |  |
| **S.Bouzid2** | n.d. | | | n.d. | | n.d. | | 72,1 | | n.d. | | n.d. | | n.d. | | n.d. | | 154 | n.d. |  |
| **S.Bouzid3** | <LOQ | | | n.d. | | n.d. | | 45,65 | | n.d. | | n.d. | | n.d. | | n.d. | | 536 | n.d. |  |
| **Sfax1** | n.d. | | | n.d. | | n.d. | | 209 | | n.d. | | n.d. | | n.d. | | n.d. | | n.d. | n.d. |  |
| **Sfax2** | n.d. | | | n.d. | | n.d. | | 161 | | n.d. | | n.d. | | n.d. | | n.d. | | n.d. | n.d. |  |
| **Sfax3** | n.d. | | | n.d. | | n.d. | | 155 | | n.d. | | 79 | | 460 | | n.d. | | n.d. | n.d. |  |
| **Tunis1** | n.d. | | | n.d. | | n.d. | | 32 | | n.d. | | n.d. | | 95 | | n.d. | | n.d. | n.d. |  |
| **Tunis2** | n.d. | | | n.d. | | n.d. | | 27,3 | | n.d. | | n.d. | | 202 | | n.d. | | n.d. | n.d. |  |
| **Tunis3** | n.d. | | | n.d. | | n.d. | | 28,15 | | n.d. | | n.d. | | 75 | | n.d. | | n.d. | n.d. |  |
| **Mahdia1** | 145 | | | n.d. | | n.d. | | 76,1 | | n.d. | | n.d. | | 640 | | n.d. | | 45 | 18 |  |
| **Mahdia2** | 124 | | | n.d. | | n.d. | | 87 | | n.d. | | n.d. | | 140 | | n.d. | | 234 | n.d. |  |
| **Mahdia3** | 151 | | | n.d. | | n.d. | | 81,5 | | n.d. | | n.d. | | n.d. | | n.d. | | n.d. | n.d. |  |

| **Conc.**  **(ngL^-1^)** | **Oxytetracycline** | **Penicillin V** | **Pipedimic acid** | **Ketoprofen** | **Acetaminophen** | **Naproxen** | **Mefenamic acid** | **Atenolol** | **CBZ** | **CBZ-epoxy** | **Caffeine** |
| --- | --- | --- | --- | --- | --- | --- | --- | --- | --- | --- | --- |
| **Gafsa1** | 46 | n.d. | n.d. | n.d. | 527000 | n.d. | 363 | 5580 | 72,4 | n.d. | 14300 |
| **Gafsa2** | 87 | n.d. | n.d. | n.d. | 1150000 | n.d. | 172 | 10100 | 64,9 | n.d. | 14400 |
| **Gafsa3** | 47 | n.d. | n.d. | n.d. | 837000 | n.d. | 267 | 6340 | 53,65 | n.d. | 14350 |
| **Monastir1** | 10 | 34 | n.d. | n.d. | 878000 | n.d. | 637 | 3730 | 195 | 14,1 | 22400 |
| **Monastir2** | n.d. | n.d. | n.d. | n.d. | 674000 | n.d. | 587 | 3150 | 187 | n.d. | 26900 |
| **Monastir3** | n.d. | n.d. | n.d. | n.d. | 726000 | n.d. | 462 | 3440 | 204,5 | n.d. | 29650 |
| **Sousse1** | n.d. | n.d. | n.d. | n.d. | 521000 | 1360 | 844 | 1390 | 608 | 64,6 | 4903 |
| **Sousse2** | 82 | n.d. | n.d. | n.d. | 569000 | 1100 | n.d. | 2371 | 541,9 | n.d. | 5527 |
| **Sousse3** | 60 | n.d. | n.d. | n.d. | 395000 | 1160 | 691 | 2704 | 536,1 | 44,9 | 5565 |
| **S.Bouzid1** | n.d. | n.d. | n.d. | n.d. | 1046544 | n.d. | 347 | 20400 | 141 | n.d. | 22500 |
| **S.Bouzid2** | n.d. | n.d. | 95 | n.d. | 930456 | n.d. | 589 | 18700 | 122 | n.d. | 23500 |
| **S.Bouzid3** | n.d. | n.d. | n.d. | n.d. | 988500 | n.d. | 468 | 20550 | 131,5 | n.d. | 21500 |
| **Sfax1** | n.d. | n.d. | n.d. | n.d. | 565000 | n.d. | 1330 | 11700 | 443 | n.d. | 20300 |
| **Sfax2** | n.d. | n.d. | n.d. | n.d. | 791000 | n.d. | 1028 | 12300 | 306 | 14,5 | 21800 |
| **Sfax3** | 26 | n.d. | 299 | n.d. | 528000 | n.d. | 1030 | 11000 | 224,5 | 27,45 | 19550 |
| **Tunis1** | n.d. | n.d. | 45 | n.d. | 1260000 | n.d. | 4959 | 12300 | 529 | n.d. | 32800 |
| **Tunis2** | 35 | n.d. | 385 | n.d. | 1220000 | n.d. | 3020 | 11100 | 406 | n.d. | 22300 |
| **Tunis3** | n.d. | n.d. | n.d. | n.d. | 1240000 | n.d. | 5416 | 11950 | 317,5 | n.d. | 27300 |
| **Mahdia1** | n.d. | n.d. | 574 | 1190 | 506000 | n.d. | 429 | 8880 | 399 | 37,4 | 36600 |
| **Mahdia2** | n.d. | n.d. | 149 | 596 | 1060000 | n.d. | 695 | n.d. | 374 | n.d. | 41200 |
| **Mahdia3** | n.d. | n.d. | 33 | 234 | 69300 | n.d. | 562 | 10760 | 371,5 | 49,2 | 38900 |

Table 5S: Octanol-water distribution coefficient (K_ow_), and water solubility at 25 ºC (S) of the selected PPCPs

| **Compound (acronym)** | **Log Kow** | **S** |  |
| --- | --- | --- | --- |
| **Benzophenone 3 (BP3)** | 3.79 | 140 mgL^−1^ |  |
| **Benzophenone 1 (BP1)** | 3.17 | 413.4 mgL^−1^ |  |
| **Benzophenone 2 (BP2)** | 2.78 | 398.5 mgL^−1^ |  |
| **4-hydroxybenzophenone (4HB)** | 3.02 | - |  |
| **4,4'-dihydroxybenzophenone (4DHB)** | 2.55 | 0.45 gL^−1^ |  |
| **2,2'-dihydroxy-4-methoxybenzophenone (DHMB)** | 3.82 | 27 mgL^−1^ |  |
| **3-(4-methylbenzylidene)camphor (4-MBC)** | 4.95 | 0.1-5.1 gL^−1^ |  |
| **Ethyl-4-*p*-aminobenzoic acid (EtPABA)** | 1.86 | 6.1 gL^−1^ |  |
| **Ethylhexyl dimethyl-4-*p*-aminobenzoic acid (ODPABA)** | 6.15 | 0.6963 mgmL^-1^ |  |
| **1H-Benzotriazol (BZT)** | 5.30 | 28 mgmL^-1^ |  |
| **5-Methyl-(1H-benzotriazol ) (MeBZT)** | 1.89 | 7 gmL^-1^ |  |
| **5,6-Dimethyl-1H-benzotriazole (DMBZT)** | 2.06 | soluble |  |
| **2-(2H-Benzotriazol-2-yl)-p-cresol (UVP)** | 4.30 | 15,37 mgmL^-1^ |  |
| **Clarithromycin (CLR)** | 0.69 | 0.33 mgL^-1^ |  |
| **Enoxacin (ENO)** | - | 3.43 gL^−1^ |  |
| **Enrofloxacin (ENR)** | 0.83 | 0.612 mgmL^-1^ | - |
| **Flumequine (FLU)** | - | 1.24 mgmL^-1^ |  |
| **Marbofloxacin (MAR)** | -1.02 | 2.6 mgmL^-1^ |  |
| **Nalidixic acid (NAX)** | 1.19 | 2.3 mgmL^-1^ |  |
| **Oxolinic acid (OXO)** | 1.43 | 1.91 mgmL^-1^ |  |
| **Oxytetracycline (OTC)** | 0.90 | 1.4 mgmL^-1^ |  |
| **Succynil-sulfathiazole (SST)** | - | 0.921 mgmL^-1^ |  |
| **Sulfadiazine (SDZ)** | 0.826 | 0.601 mgmL^-1^ |  |
| **N^4^-acetylsulfadiazidine (acSDZ)** | - | 0.601 mgmL^-1^ |  |
| **Sulfamerazine (SMR)** | 1.41 | 0.304 mgmL^-1^ |  |
| **N^4^-acetylsulfamerazine (acSMR)** | - | - |  |
| **N^4^-acetylsulfamethazine (acSMZ)** | - | - |  |
| **Sulfamethoxazole (SMX)** | 0.27 | 0.459 mgmL^-1^ |  |
| **N^4^-acetylsulfamethoxazole (acSMX)** | - | - |  |
| **Sulfamethoxypyridazine (SMP)** | 0.32 | 0.325 mgmL^-1^ |  |
| **Sulfapyridine (SPY)** | 0.05 | 0.235 mgmL^-1^ |  |
| **N^4^-acetylsulfapyridine (acSPY)** | - | - |  |
| **Sulfaquinoxaline (SQX)** | 1.7 | 0.0761 mgmL^-1^ |  |
| **Sulfathiazole (STZ)** | 0.91 | 0.921 mgmL^-1^ |  |
| **Sulfisomidin (SSM)** | - | 0.229 mgmL^-1^ |  |
| **Sulfadimethoxine (SDM)** | 0.16 | 0.278 mgmL^-1^ |  |
| **Sulfanitran (SNT)** | - | 0.0343 mgmL^-1^ |  |
| **Sulfabenzamide (SBD)** | - | 0.134 mgmL^-1^ |  |
| **Trimethoprim (TMP)** | 4.77 | 0.615 mgmL^-1^ |  |
| **Penicillin V (PEN)** | 1.83 | 0.454 mgmL^-1^ |  |
| **Pipemidic acid (PIP)** | -2.1 | 0.746 mgmL^-1^ |  |
| **Paracetamol (AMP)** | -0.39 | 4.15 mgmL^-1^ |  |
| **Atenolol (ATL)** | 0.61 | 0.429 mgmL^-1^ |  |
| **Ketoprofen (KEP)** | 0.97 | 0.0213 mgmL^-1^ |  |
| **Mefenamic acid (MFA)** | 5.12 | 0.0137 mgmL^-1^ |  |
| **Naproxen (NAP)** | 3.18 | 0.0511 mgmL^-1^ |  |
| **Carbamazepine (CBZ)** | 2.23 | 0.152 mgmL^-1^ |  |
| **Carbamazepine 10,11-epoxy (CBZ-epoxy))** | - | - |  |
| **Ofloxacin (OFL)** | 1.41 | 1.44 mgmL^-1^ |  |
| **Caffeine (CAF)** | 0.98 | 11.0 mgmL^-1^ |  |
|  |  |  |  |

Table S6: Environmental Predicted No Effect Concentration (PNEC-ENV), extracted from literature (Orias and Perrodin 2013).

| **Compounds** | **PNEC-ENV (µgL^-1^)** |
| --- | --- |
| Ciprofloxacin | 0.45 |
| Enrofloxacin | 1.9 |
| Ofloxacin | 10 |
| Orbifloxacin | 259.2 |
| Clindamycin | 0.1 |
| Azithromycin | 0.02 |
| Clarithromycin | 0.08 |
| Trimethoprim | 100 |
| Ampicillin | 0.87 |
| Nalidixic acid | 0.072 |
| Oxolinic acid | 0.0073 |
| Pipemidic acid | 447 |
| Sulfamethoxazole | 0.6 |
| Sulfapyridine | 6.2 |
| Tetracycline | 3.2 |
| Amoxicillin | 0.250 |
| Azithromycin | 0.250 |
| Ciprofloxacin | 0.064 |
| Trimethoprim | 0.500 |
| Oxytetracycline | 0.500 |
| Marbofloxacin | 0.01 |
| Flumequine | 0.01 |
| Sulfabenzamide | 0.000071 |
| Sulfadimethoxine | 0.24 |
| Sulfamerazine | 0.68 |
| Acetylsulfapyridine | n.a. |
| Sulfadiazine | 0.6 |
| Ketoprofen | 2 |
| Mefenamic acid | 3.9 |
| Acetaminophen | 6.92 |
| Atenolol | 20 |
| Carbamazepine | 2 |
| Caffeine | 0.00005 |
